# Integrating Wheat Nucleolus Structure and Function: Variation in the Wheat Ribosomal RNA and Protein Genes

**DOI:** 10.3389/fpls.2021.686586

**Published:** 2021-12-24

**Authors:** Rudi Appels, Penghao Wang, Shahidul Islam

**Affiliations:** ^1^AgriBio, Centre for AgriBioscience, La Trobe University, Bundoora, VIC, Australia; ^2^Faculty of Veterinary and Agricultural Science, Melbourne, VIC, Australia; ^3^School of Veterinary and Life Sciences, Murdoch University, Murdoch, WA, Australia; ^4^Centre for Crop Innovation, Food Futures Institute, Murdoch University, Murdoch, WA, Australia

**Keywords:** nucleolar dominance, rRNA structure, ribosomal protein (RP), sequence variation, associated phenotypes

## Abstract

We review the coordinated production and integration of the RNA (ribosomal RNA, rRNA) and protein (ribosomal protein, RP) components of wheat cytoplasmic ribosomes in response to changes in genetic constitution, biotic and abiotic stresses. The components examined are highly conserved and identified with reference to model systems such as human, Arabidopsis, and rice, but have sufficient levels of differences in their DNA and amino acid sequences to form fingerprints or gene haplotypes that provide new markers to associate with phenotype variation. Specifically, it is argued that populations of ribosomes within a cell can comprise distinct complements of rRNA and RPs to form units with unique functionalities. The unique functionalities of ribosome populations within a cell can become central in situations of stress where they may preferentially translate mRNAs coding for proteins better suited to contributing to survival of the cell. In model systems where this concept has been developed, the engagement of initiation factors and elongation factors to account for variation in the translation machinery of the cell in response to stresses provided the precedents. The polyploid nature of wheat adds extra variation at each step of the synthesis and assembly of the rRNAs and RPs which can, as a result, potentially enhance its response to changing environments and disease threats.

## Introduction

The wheat seed, like all plant seeds, is a “special living state” retaining only 10–15% moisture in the “dry” state to which tissue in the grain has adapted for long-term storage (Bonner and Varner, 1965; Swift and O’Brien, 1972). The dry scutellum/embryo (=“wheat germ”) is a source of viable ribosomes for the translation of stored messenger RNA when the seed rehydrates, and historically the wheat germ was an early source of ribosomes for the *in vitro* translation of isolated messenger RNA (Armache et al., 2010). In this review, the overall 3D structure of wheat ribosomes determined by Armache et al. (2010) is used as a basis for reviewing the component RNA and proteins in wheat in order to understand changes in ribosome structure and the adaptation of the translational process in cells to biotic and abiotic stresses.

The RNA components of wheat cytoplasmic ribosomes (rRNA) are encoded by very large tandem arrays of gene units that are transcribed by a dedicated RNA polymerase (RNApol1) within a compartment of the nucleus called the nucleolus (Appels and Dvorak, 1982; Barker et al., 1988; Thompson and Flavell, 1988; Handa et al., 2018; Tulpová et al., 2020). The hexaploid nature of wheat, comprising seven chromosome pairs in the A, B, and D genomes, means that there exists a complex set of interactions between the genes coding for the rRNA and ribosomal proteins (RPs) to generate flexibility in the composition of ribosomes. An estimated 42–100 Mb of the genome is devoted to coding for rRNA and, at this mega-level, structural differences between the major rDNA loci on chromosomes 1B and 6B are argued to be important in the autoregulation of rDNA expression and the silencing of minor rDNA arrays. Accompanying the production of rRNA, a total of 170 proteins have been assigned (high confidence, HC) to the cytoplasmic ribosome subunits 40S and 60S, and organelle subunits 30S and 50S in wheat proteome studies (Ford et al., 2011; Duncan et al., 2017; Islam et al., 2020). The coordinated production and integration of both RNA and protein components into the wheat cytoplasmic ribosome assembly processes are considered in the present review in the context of adjustment to selection pressures and response to biotic and abiotic stresses. The compilation of protein sequences in this review focused on wheat *per se* and entries from Arabidopsis and rice. UniProt identifiers were used to recover amino acid sequences for searches against the *Triticum aestivum* L. reference genome using BLASTP in Ensembl^1^ and manual recovery of low-confidence (LC) gene models in a genome viewer Apollo instance^2^ for the Chinese Spring wheat genome assembly ver1 to curate their status. The predicted gene models were confirmed using the Phyre2^3^ 3D structure predictions for domains and inspecting protein domains in Pfam^4^ and InterPro^5^ databases. Coordinates for the Traes gene IDs from Ensembl Plants were used to locate the gene models in the CS wheat genome ver1 assembly using the Apollo instance for the wheat CS genome assembly ver1 noted above with the gene models curated by the alignments to RNA-seq from the standard tissues, grain, leaf, roots, stem, and spike (Supplementary Table 1). During the course of producing this review, version 2.1 of the wheat Chinese Spring genome was published (Zhu et al., 2021) and although version 1 gene identifiers are used in the present manuscript, Supplementary Table 2 provides a cross-reference of the version 1 gene identifiers to the version 2.1 gene identifiers that will appear in Ensembl Plants updates in due course.

## The rDNA Genome Regions in Wheat Nuclei

**FIGURE 1:**
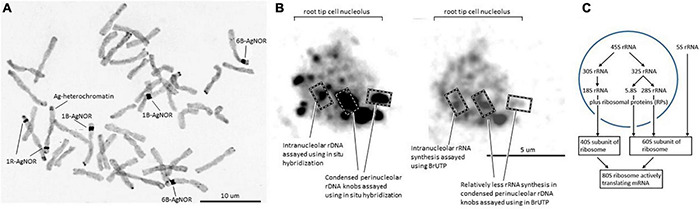
**(A)** Metaphase chromosomes from a wheat line with a 1R disomic addition (modified from Silva et al., 2008, doi.org/10.1371/journal.pone.0003824.g002). Dark staining regions corresponding to the nucleolar organizers are indicated as AgNOR; other dark staining regions of the rye chromosome correspond to heterochromatin/C-banding regions (marked as Ag-heterochromatin). **(B)** Wheat nucleolus, modified from Silva et al. (2008, doi.org/10.1371/journal.pone.0003824.g002) and Correll et al. (2019, doi.org/10.3390/cells8080869). In the left panel, dark regions are the condensed chromatin regions with rDNA as assayed by the wheat clone pTa71 (Gerlach and Bedbrook, 1979) as a probe for *in situ* hybridization. Silva et al. (2008) defined intranuclear dots housing the more dispersed rDNA that is more active in transcription (examples indicated) and perinucleolar knobs housing the condensed rDNA that was relatively inactive in transcription. The distribution of newly synthesized RNA in the right panel is distributed throughout the nucleolus as measured by the incorporation of labeled UTP (BrUTP). **(C)** Summary of the flow of RNA processing, and assembly into the mature cytoplasmic small ribosomal subunit (40S) and the large subunit (60S), to form the active 80S ribosome, including the independent production of 5S rRNA.

**FIGURE 2:**
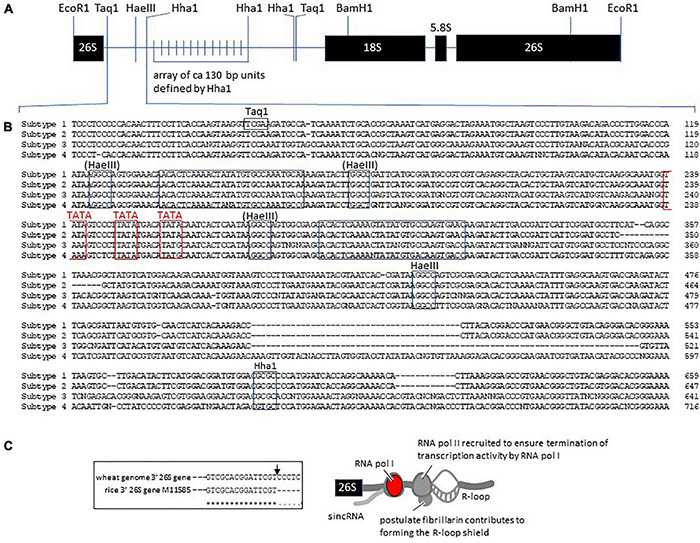
Termination of rDNA transcription and the engagement of RNA polymerase II to ensure efficient rRNA production. **(A)** Summary of the 9,000 bp rDNA unit in wheat. The 26S gene-3′ downstream is followed by the repetitive region of ca. 130 bp units leading to the promoter region and start of transcription (see Supplementary Figure 3) in the 18S gene 5′ upstream region. **(B)** The 26S gene-3′ downstream ca. 650 bp that distinguishes the S1–S4 subtypes of rDNA in wheat. The *Taq*1, *Hae*III, and *Hha*1 that could be related to the structure defined for the clone pTa250 by Appels and Dvorak (1982) are boxed. The (*Hae*III) sites were not identified in pTa250. The TATA box regions are emphasized in red and are postulated to engage the TATA box-binding protein (TBP) for contributing to engaging RNA polymerase II in forming an R-loop shield. **(C)** Model for the protein complexes formed in the 26S gene-3′ downstream region to ensure RNA pol I transcription is properly terminated, based on the studies on human rDNA structure and function (Fomproix et al., 1998; Abraham et al., 2020; doi.org/10.1038/s41586-020-2497-0). The prominent nucleolar proteins, nucleolin and fibrillarin, are possibly involved as discussed in the earlier text (Nasirudin et al., 2004; Pontvianne et al., 2007; Rodriguez-Corona et al., 2015). The inset (boxed) indicates the 3′-end of the rice 26S RNA gene to confirm the 3′-end of the wheat 26S RNA gene, according to Armache et al. (2010).

The nucleolus organizer region (NOR) is a classical feature of metaphase chromosomes where major rDNA loci are visible as so-called secondary constrictions in the respective chromosomes (Figure 1A; Silva et al., 2008) in addition to the primary constriction (centromere) visible in all chromosomes. In Figure 1, silver (Ag) staining was used to visualize the location of the NOR because it detects concentrations of nucleic acid molecules and argyrophilic Ag-binding proteins and provides a sensitive assay for the NOR and associated rDNA transcription (Silva et al., 2008) in both plants and animals (Figure 1). The Ag staining assays a set of argyrophilic protein markers associated with active ribosomal genes (AgNOR proteins) that reduce and thus deposit the Ag under cytochemically acidic conditions in NORs of metaphase chromosomes and nucleoli of interphase nuclei in fixed tissue (Sirri et al., 2000; Caperta et al., 2002). Studies in model systems have identified nucleolin and nucleophosmin as major AgNOR proteins in the nucleolus with smaller contributions coming from RNA polymerase 1 (RNA pol I) subunits and the transcription factor UBF/UAF30 (Sirri et al., 2000; see also Figure 2 and Supplementary Table 1 for wheat homologs). The wheat models for nucleophosmin show a very clear acidic amino acid cluster feature considered to be characteristic for Ag binding, DDLMKNNFGVEGDEDDEDDDEDED, in the C-terminal region (Sirri et al., 2000).

Nucleolus organizer regions are generally considered to feature three subcompartments, namely pale staining structures fibrillar centers (FCs) comprised of fine fibrils, a surrounding densely stained fibrillar component (DFC), and a granular component (GC) in which the FC and DFC are embedded. Given the fundamental nature of ribosome production, it seems reasonable to adopt this model of the nucleolus established in human studies, for wheat, and to envision transcription of the rDNA occurring at the interface between the FCs and the DFC with nascent transcripts and pre-ribosomes progressively migrating from the DFC to the GC (Thiry and Lafontaine, 2005). The high concentration of a limited set of nucleic acid molecules plus associated proteins and rDNA chromatin are considered to form the nucleolus as a phase separated feature within the nucleus not bound by membranes (concept developed in model systems, Berry et al., 2015). Within the nucleolus in wheat, the ribosomal chromatin is located in interphase nuclei as condensed perinucleolar chromatin knobs varying between 3 and 4 μm, and intranucleolar condensed dots ranging from 1 to 2 μm in diameter (Figure 1B; Silva et al., 2008). Variation involving the relative distribution of rDNA in the intranucleolar dots and perinucleolar knobs is found. The long arrays of rDNA units that are not transcribed into rRNA are generally located in condensed chromatin (Figure 1B). The long arrays of rDNA active chromatin are distributed across most of the nucleolus based on the dispersed distribution of newly synthesized RNA (Figure 1B). The 45S rRNA precursor that is initially formed interacts with RPs and assembly factors (AFs) in the nucleolar space for processing to the 18S, 5.8S, and 26S rRNAs, the foundations for the ribosome subunits (Figure 1C).

The 5SrRNA indicated in Figure 1C is produced from independent loci composed of tandem arrays of short units (120-bp gene sequence, intergenic spacer ca. 280 bp, Supplementary Figure 2) predominantly in the satellite region of chromosome 1BS and smaller numbers on 1DS, and tandem arrays of long units (intergenic spacer ca. 380 bp) predominantly on 5BS with smaller numbers on 5AS and 5DS (Dvorak et al., 1989; Reddy and Appels, 1989; Baum and Bailey, 2001; Sergeeva et al., 2017). Genome arrays for the 5SrRNA gene units on 5BS have been shown to be in uninterrupted tandem arrays of the long units, and lower numbers of these long units in clusters interrupted by inserts of mobile elements (Sergeeva et al., 2017).

The genome regions encoding rRNA components at the NORs are a challenge in genome assemblies because of the extreme length of the repetitive arrays of near identical gene units and only recently optical mapping has provided a clear view of the tandem arrays in wheat (Tulpová et al., 2020). In the human genome studies, some full and partial arrays of 18S-5.8S-28S rDNA units are assembled on each of the five acrocentric p-arms, but the centers of these arrays are currently represented by a total of 11.5 Mbp of unknown sequence (Ns) in the assemblies on chromosomes 13, 14, 15, 21, and 22. The arrays are near-identical tandem repeats, and so while the content of these arrays is known, the variation in substructure within the arrays remains to be determined. A detailed study of the 18S-5.8S-28S rDNA units on human chromosome 21 succeeded in assembling long arrays (Kim et al., 2018) and revealed heterogeneity at the single locus for rDNA units on this chromosome suggesting the possibility that this variation may relate to the dynamics of ribosome function. In wheat, the application of optical mapping on high molecular weight DNA isolated from flow sorted chromosome arms physically identified the arrays of 2,813 units on 6BS (26.87 Mb), 1,378 (12.96 Mb) on 1BS, and 170 (1.63 Mb) on 5D. A small number of complete units were identified on 1AS (29 units, 0.43 Mb). The optical mapping thus provided a minimum estimate of 4,390 units (42 Mb) in tandem arrays, within the wheat cv Chinese Spring (CS) genome, but does not allow for rDNA fragments external to the tandem arrays and dispersed in the genome. The latter most likely contribute to the higher numbers of total rDNA units reported by Handa et al. (2018). Similar to some human rDNA arrays, the rDNA arrays on chromosome 6BS of wheat showed heterogeneity due to interspersion of non-rDNA sequences in contrast to the relatively homogeneous arrays on 1BS and 5DS (Handa et al., 2018; Tulpová et al., 2020). Studies in wheat are generally agreed on the relative proportions of rDNA units on 1BS (31%), 6BS (61%), and 5DS (8%) (Flavell and O’Dell, 1976; Handa et al., 2018; Tulpová et al., 2020).

Assembly factors initiate the formation of small and large “preribosomal” subunits that accumulate in the GC. The AgNOR proteins, such as nucleolin and nucleophosmin (Figure 1), appear to be within the network of AFs active in the ribosome assembly process based on their capacity to bind RNA. Nucleolin is an RNA-binding protein in the nucleoli of all eukaryotes, and in plants it has been well studied at the structure-function level in Arabidopsis, rice (Pontvianne et al., 2007), and pea (Nasirudin et al., 2004). In wheat, the nucleolin gene models are located on chromosomes 2A, 2D, 5D, 7A, 7B, and 7D and display RNA-binding domains in Pfam (RMM domains, Supplementary Figure 1) and Phyre2 analyses (Kelley et al., 2015; predicted fold c6r5kH). The C-terminal glycine-rich region is homologous to the region assigned to have helicase attributes as reported by Nasirudin et al. (2004) and has a number of differences between the wheat gene models (Supplementary Figure 1). Based on RNA-seq alignments, all the homologs are highly expressed in the standard, root, leaf, stem, spike, and grain tissues assayed except for the 5D homolog, which has a low-to-moderate expression. The N- and C-terminal regions of the nucleolins are the main source of single amino acid differences between the gene models, with the 5D nucleolin having an additional major difference in missing the entire C-terminal glycine-rich domain and seem to be a pseudogene. The 2A and 2D wheat gene models have 19 exons, and the 7A, 7B, and 7D gene models have 13 exons, similar to the distinction between Arabidopsis AtNuc-L2 (18 exons, At3g18610) and AtNuc-L1 (15 exons, At1g48920) nucleolins. Comparisons between the genomic regions of wheat varieties available in the genome viewer DAWN (Watson-Haigh et al., 2018) for the wheat nucleolins indicated 50 positions in the coding regions of the genes (CDS) were captured as variable positions assayed as single nucleotide polymorphisms (SNPs) at the DNA sequence level. In the wheat varieties inspected for variation in the nucleolin gene region, a total of nine of the variable positions in the genome sequence also showed differences at the amino acid sequence level between the homologous gene models of the reference genome *per se* (Supplementary Figure 1). An additional set of nine amino acid sequence differences between the 2A and 2D nucleolins and the 7A, 7B, and 7D nucleolins of the reference genome were not captured as SNPs at the genome level in the wheat varieties set available in DAWN.

Mutation studies in Arabidopsis (Pontvianne et al., 2007) emphasize the fundamental importance of nucleolin in plant growth and development, and for wheat the alignment of the 2A and 2D nucleolin gene models indicates the conserved domains (RMM, RNA-binding domains, Supplementary Figure 1) that actually show relatively high levels of polymorphisms at the genome level in terms of the distribution of SNPs. The glycine-rich region at the C-terminal end of the protein shows variation between the 2A and 2D nucleolin gene models, this has not been captured by variation in breeding programs as judged from inspecting varieties available in the DAWN viewer (Supplementary Figure 1). The combination of changes at the amino acid and SNP levels can be considered as gene haplotypes and can be viewed as defining fingerprints that could provide markers for associating rRNA loci that are preferentially expressed in terms of the phenomenon of nucleolar dominance described below or in response to environmental stress. The concept of gene haplotypes (broad sense as per Wilhelm et al., 2013) providing function-based markers for associating particular ribosome-related protein (RPs) variants with responses in translation activity of the ribosomes to stress is considered further (later) as we establish a haplotype dictionary for the RPs in wheat.

The fibrillarin protein is another major component of the nucleolus (Rodriguez-Corona et al., 2015) that co-localizes with AgNOR proteins located in the DFC and FC in model systems. The fibrillarin gene is conserved in model plants and animals and thus allows the wheat homologs to be identified on chromosomes 6A, 6B, 6D, 7A, 7B, and 7D. As was the case for the nucleolin gene homologs, RNA-seq alignments indicate that all the fibrillarin homologs are highly expressed in the standard, root, leaf, stem, spike, and grain tissues assayed.

**FIGURE 3:**
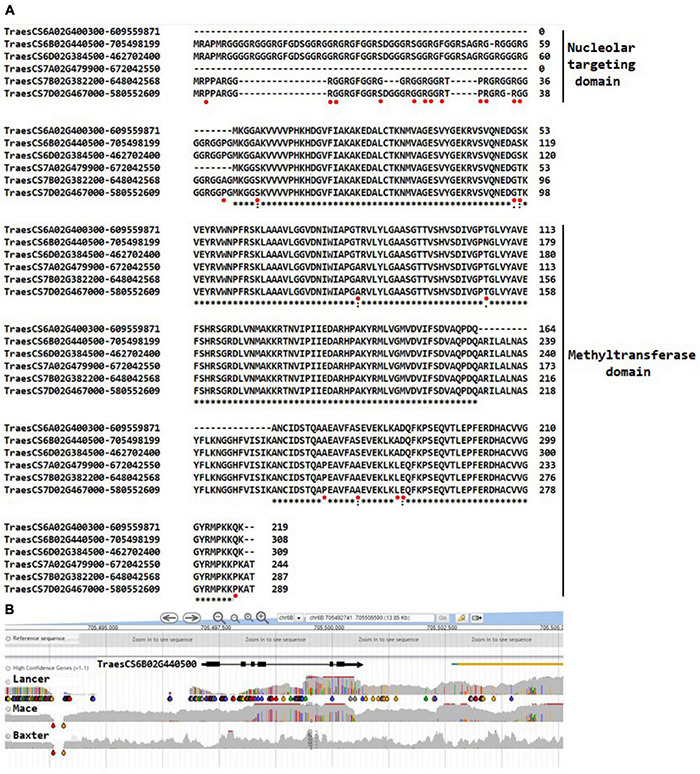
**(A)** The wheat fibrillarin genes on chromosomes 6A, 6B 6D, 7A, 7B, and 7D. Sequences from Arabidopsis and rice were downloaded, and UniProt identifiers were used to recover amino acid sequences for searches against the *Triticum aestivum* L. genomes using BLASTP in Ensembl (http://plants.ensembl.org/index.html). Alignment at the amino acid level to validate the identification on the wheat gene models against the well-characterized Arabidopsis AAF00542 gene; the fibrillarin TraesCS6D02G462702400 had an identical amino acid sequence to the fibrillarins from human and yeast. The methyltransferase domain is well conserved in contrast to the nucleolar-targeting domain, which shows relatively more diversity. The * indicates the same amino acid is at the respective positions, and spaces and dots indicate amino acid change. The red spots indicate the positions of the single nucleotide polymorphisms (SNPs) in an assessment of wheat varieties available in the viewer DAWN. **(B)** A representative view of the SNP diversity at the DNA sequence level identifying gene haplotypes for TraesCS6B02G440500 using wheat varieties Lancer, Mace, and Baxter as examples. Lancer and Mace have been sequenced in the 10 genome project (Walkowiak et al., 2020). The gray areas indicate the variable genome coverage of the available sequence data, and the colored “drops” identify positions in the sequence that are uniformly changed from that of the reference Chinese Spring genome sequence (orange = change to G; red = change to T; green = change to A; blue = change to C). The SNP analysis was possible using DAWN (Watson-Haigh et al., 2018; http://crobiad.agwine.adelaide.edu.au/dawn/jbrowse/). The DAWN viewer uses standard genome format and can show the location of SNP at the genome sequence level.

The alignments for fibrillarin shown in Figure 3A complement the observations for nucleolin in identifying gene haplotypes that can provide functional markers for associating protein variants with plant phenotypes. The N-terminal glycine-rich parts of the predicted fibrillarins are particularly variable and are actually absent from the 6A and 7A gene models. Since this part of the molecule is responsible for targeting the protein to the nucleolus as suggested by mutation studies in Arabidopsis (review, Rodriguez-Corona et al., 2015), the structural variation within wheat suggests that a considerable flexibility exists for delivering the methyltransferase activity required for modifying rRNA. As is evident in Figure 3B, the fibrillarin gene models in wheat show clear haplotype differences between wheat varieties based on the inspection of varieties available in the genome viewer DAWN (Watson-Haigh et al., 2018). The haplotypes are defined by differential SNP distributions. The representative example in Figure 3B shows exon 1 in particular to have a clear haplotype difference between the wheat varieties Mace and Lancer, and since this exon encodes the nucleolar-targeting region for fibrillarin, it raises the possibility of associating this genome difference with phenotypes that differentiate wheat cultivars. The association would be considered in the context that fibrillarin is biologically an essential protein (Loza-Muller et al., 2015), well known as a molecular marker of transcriptionally active RNA polymerase. Fibrillarin methyltransferase activity is argued to be the primary methyltransferase for methylated sites early in preribosomal processing and subsequent structural ribosome stability (Rodriguez-Corona et al., 2015). Consistent with this role for fibrillarin in rRNA synthesis, Tessarz et al. (2014) demonstrated that methylation of Q105 or a substitution Q105A in histone 2A by fibrillarin in human and yeast cells specifically increased rRNA synthesis. In wheat, Q105 is substituted Q105H in the same H2A sequence segment and has a Q98 in the same sequence segment where human H2A has a D genome so although the fibrillarins are identical in wheat, human, and yeast, a direct parallel for the effects of H2A methylation needs further experimental work.

## Variation in the Nucleolar Dominance Phenomenon in Wheat

The diversity in major nucleolar proteins, such as nucleolin and fibrillarin, may inform the well-studied phenomenon of nucleolar dominance. Observations on rDNA transcription in the nucleoli of wheat lines with and without a rye chromosome (1R, the major source of rDNA in rye, Silva et al., 2008) or 1U from *Aegilops umbellulata* (Flavell et al., 1988) have indicated that different sources of rDNA regions moderate the expression of each other. Similarly within wheat *per se*, Handa et al. (2018) identified four rDNA unit subtypes (S1–S4) based on differences within the 3′ transcribed spacer regions in Nor-B1 (on 1BS) and Nor-B2 (on 6BS), Figure 2, and demonstrated by quantitative PCR that S1 subunits were predominantly expressed. The S2 subunits were relatively more abundant, but only weakly expressed. Overall, 31.4 and 64.1% of the rDNA units have been assigned to the major NORs in 1BS and 6BS, respectively (Tulpová et al., 2020). The minor loci in 5DS and 1AS have 3.9 and 0.7% of the rDNA units, respectively. The expression of S3 subunits on 5DS increased in the ditelosomic genetic stocks Dt1BL (1BS missing) and Dt6BL (6BS missing), suggesting that S3 is subjected to the chromosome-mediated silencing. In the context of the differential distribution of rDNA in the condensed chromatin (Figure 1B), Handa et al. (2018) found genome regions adjacent and distal to the major NORs were expanded compared to homologous regions on 1A, 1D, 6A, and 6D, where rDNA loci are no longer present. Handa et al. (2018) suggested that these regions flanking the rDNA loci on chromosomes 1B and 6B could be a potential source for distinguishing the respective rDNA regions for macro (chromatin)-level condensation and render their rDNA units transcriptionally inactive. Similar models based on modifying the chromosome structure around the rDNA units on the *Drosophila* X chromosome have been developed to account for heterochromatin modifying the relative expression of rDNA on the X and Y chromosomes (Hilliker and Appels, 1982).

In wheat, the “non-syntenic” regions of 5–12 Mb of DNA flanking the 1B and 6B rDNA regions are mainly distinguished by a relatively higher transposable element content (Handa et al., 2018) that is often associated with inactive chromatin. At this macro-level and complementing, the cytological observations in Figure 1B, the degree of condensation of rDNA chromatin has been assayed using the sensitivity of rDNA to the enzyme DNAase I in isolated nuclei (Thompson and Flavell, 1988). The DNAase I sensitivity assays led to the conclusion that the promoter regions of some wheat rRNA genes possess a more accessible chromatin structure, with the proportion of hypersensitive genes in a NOR argued to be related to observed activity. The genes that displayed hypersensitive DNase I sites were preferentially non-methylated at CCGG sites in the intergenic spacer immediately preceding the promoter. Thus, the chromatin structure around the promoter of active rRNA genes was differentiated from that in transcriptionally inactive genes and correlated with changes in cytosine methylation. In the case of the wheat-1U addition line studied by Thompson and Flavell (1988), the affinity for (predicted) factors within the DFC/FC interface to assign rDNA units to active transcription is 1U > 1B > 6B. Similarly within wheat *per se*, the additional identification of the S1–S4 rDNA subtypes, and SNP-based haplo-subtypes (Tulpová et al., 2020), is consistent with the preferential recruitment of rDNA units into an active state having a structural basis. Quantitative differences in transposable element levels both within the 6B NOR rDNA arrays (Tulpová et al., 2020) and the regions flanking the 6B NOR could in principle account for the lower transcription of the 6B rDNA genes if the 1B and 6B NORs were competing for limited sites for condensation within the FC, leaving relatively more 1B rDNA for transcription at the DFC/FC interface. A competition model would account for changes in the source of NORs utilized for producing rRNA, depending on the different NORs present in the genetic makeup of the wheat analyzed. Consistent with “non-syntenic” 6B regions as drivers for differentiating the 6B NOR from the 1B NOR is the finding by Handa et al. (2018) that this region is characterized by higher levels of the histone methylation mark, H3K27me3, a chromatin feature that is generally associated with a condensed/gene repressed state of chromatin. The finding that hypersensitive DNase sites included CCGG sites in the intergenic spacer immediately preceding the promoter were preferentially non-methylated in the rDNA units from the 1B NOR compared to the 6B NOR (Thompson and Flavell, 1988) is also consistent with a chromatin structure-based differentiation of the 1B and 6B NOR loci.

Although the structure of the S1–S4 subtypes of rDNA units within the rDNA arrays in wheat NORs could not be extended beyond the 26S gene-3′downstream region shown in Figure 2 due to ambiguities in recovering experimental chimeric sequences (artifacts) in genome assemblies (H. Handa, personal communication), it is evident that more sequence variation is observed in the so-called non-transcribed spacer (NTS) regions further from the 26S gene-3′downstream region. Extensive polymorphism in the repetitive sequence region comprised of 120–130 bp units within the NTS is well established (Appels and Dvorak, 1982; Lassner et al., 1987; Lagudah et al., 1991) at the level of the number of repetitive units within the NTS and at the DNA sequence level. Duplicated sequences dominate NTS variation and Lassner et al. (1987) identified a consensus sequence of CACGTACACGGA as a signature and basis for the range of variation found, suggesting that the sequence possibly provides sites for within locus recombination or DNA replication slippage events.

In terms of sequence variation within the genes coding for the 18S, 5.8S, and 26S rRNA genes, Tulpová et al. (2020) identified pairs of SNPs for consensus 26S gene sequences from each of the 1BS, 6BS, and 5DS NOR loci that defined unique 26S gene haplotypes. The fact that the 26S gene haplotypes could be defined indicated that there was little if any genetic exchange between the NOR loci even though the long tandem arrays were similar in sequence. This finding was consistent with the conclusions by Lassner et al. (1987) from their sequence comparisons of rDNA clones from the B and D genomes of wheat. Interestingly, the 26S gene haplotypes also allowed the source of rDNA transcripts to be identified in different tissues, and this showed that RNA from mature leaf had the lowest proportion of 6BS transcripts relative to root tip and coleoptile samples. In seeds some unassigned transcripts were found. The possibility that the lower amount of 6B 26S rRNA in the leaf tissue was due to RNA undergoing a faster turnover in this tissue was raised by Tulpová et al. (2020). Consistent with the observations in wheat, the detailed analysis of the structure of tandem rDNA units at Arabidopsis NOR loci (Sims et al., 2021) indicated a clustering of variants that could be traced by SNP haplotypes for the respective 26S rRNA genes. The Arabidopsis study also indicated that ribosomal variants showed tissue-specific expression as well responses to certain stress conditions.

## Transcription of 18S-5.8S-26SrDNA and 5SrDNA

The RNA polymerases essential for rRNA synthesis are RNA polymerase I (for 18S, 5.8S, and 26S RNAs) and RNA polymerase III (for 5S RNA, see Supplementary Figure 2). Unlike some of the RNA pol II subunits, none of the subunits defined in Arabidopsis by Ream et al. (2015) are sufficiently conserved to allow homologs to be identified in the wheat or rice genomes. The subunits shared between RNA pol I, pol II, and pol III (Rpb5, Rpb6, Rpb8, Rpb10, and Rpb12) for RNA pol I do not give clear homologs even though the respective subunits for RNA pol II (from Arabidopsis) can identify homologs in wheat and rice, and this suggests that the boundaries of conservation are not as constrained for RNA pol I and III as they are for RNA pol II. At the genome sequence, there are features of the wheat intergenic rDNA region that can relate to aspects of the controls operating on RNA pol I found in studies of human rDNA transcription (Abraham et al., 2020). In human rDNA transcription, the 3′ region downstream from the 26S rDNA unit has been identified as a point of engagement of RNA pol II in controlling rRNAs expression. The authors argued that RNA pol II generated structures known as R-loops in the intergenic spacers flanking nucleolar rRNA genes (Figure 2) and prevented RNA pol I from producing sense intergenic non-coding RNAs (sincRNA) that could disrupt nucleolar organization and rRNA expression. In this context, it is possible that the finding by Handa et al. (2018) of sequence differences in the 26S gene 3′ downstream region in wheat (Figures 2A,B) may relate more directly to influencing rDNA transcription activity depending on the efficiency of R-loop formation and associated variation in the activity of RNA pol I. In Figure 2C, the structural features of the S1–S4 subtype 26S gene-3′downstream region defined by Handa et al. (2018) are interpreted in the context of RNA pol II engagement in highlighting the prominence of the TATA motifs (classically core elements of RNA pol II promoters) in these regions. Major nucleolus proteins, such as the nucleolin and fibrillarin gene models in wheat (Supplementary Figure 1), have RNA-interacting domains that could also contribute to facilitating the establishment of a proposed R-loop shield. Rodriguez-Corona et al. (2015) noted that the rDNA transcription instability in permeabilized mammalian tissue culture cells infused with fibrillarin antibodies (Fomproix et al., 1998) may be due to the antibodies preventing fibrillarin from contributing to the R-loop shield, which normally blocks sense sincRNA formation by RNA pol I (Figure 2).

At the end of the NTS, downstream from the 26S gene 3′-downstream region is the promoter region for forming the correct RNA pol I initiation complex at − 1 to approximately − 200 bp upstream from the start of transcription (Lassner et al., 1987; Supplementary Figure 3). The region shows the sequence features of AT and GC clusters that are well characterized for the core promoter region in model systems such as AATGGGGG^–20^CTAAAACCTC^–10^GGGTATAGT^–1^ (TATA box underlined), and further upstream for the binding site for the upstream activity factor (UAF), referred to as the upstream-control-element region (G^–200^GTCCGGGAGA^–190^AAAAAAGGCC^–180^; Pisl and Engel, 2020). The TBP and Rrn3 (TF-A1) factors are two significant components that direct RNA pol I into the initiation complex, and they are sufficiently well conserved to allow wheat homologs to be identified using the bioinformatics identification pipeline documented earlier in this review. The wheat homologs include TraesCS1B02G151700 (TBP-1), TraesCS5A02G022000 (TBP-2), TraesCS5B02G018500 (TBP-2), TraesCS5D02G027800 (TBP-2), TraesCS6A02G171400 (Rrn3), and TraesCS6D02G161100 (Rrn3); see also Supplementary Table 1. Another significant component for directing RNA pol I to the promoter is the upstream activity factor (UAF, UBF), which belongs to a large family of transcription control proteins characterized by the SWIB/MDM2 domain associated with proteins involved in chromatin remodeling. The wheat homologs could be identified using the yeast UAF30 as a reference (Iida and Kobayashi, 2019) and had the predicted fold of d1v31a in Phyre2 (Kelley et al., 2015) for UAF30. The wheat homologs were found on chromosomes 2A and 2B, TraesCS2A02G488300, TraesCS2B02G515900, and chromosome 7D, TraesCS7D02G242300; see also Supplementary Table 1. Inspection of the genome sequence indicated homologous gene models also existed on 2D, 7A, and 7B, but gaps in the genome sequence prevented unambiguous identification. One of the AFs in the spatial and temporal coordination of rRNA production in model systems is the factor Rrp5 (Khoshnevis et al., 2019), and HC gene models were located on chromosomes 1A, 1B, and 1D. The wheat Rrp5 gene models were confirmed based on the presence of a domain covering 14–15% of the CDS sharing a predicted 3D structure, c5c9sB in Phyre2, and were assigned to the gene models TraesCS1A02G06730, TraesCS1B02G085800, and TraesCS1D02G068300; see also Supplementary Table 1. A striking feature for all gene models likely to represent factors involved in the different levels of rRNA production is the variation that exists for the gene models in the wheat genome, as was found for nucleolin and fibrillarin. The variation exists at the amino acid sequence level between homologous members of a locus and at a broader genome sequence level where gene haplotypes for SNPs are clear between wheat varieties analyzed for SNPs relative to the reference genome of Chinese Spring ver 1.0 in DAWN (see Supplementary Figure 4 for the Rrp5 example).

**FIGURE 4:**
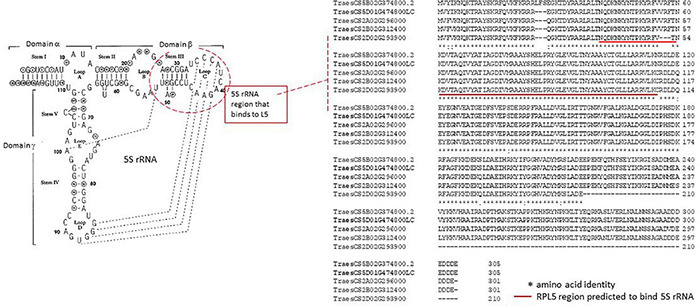
Structure of wheat 5S rRNA and the ribosomal L5 protein (RPL5) from wheat. The consensus 5S rRNA sequence and secondary structure modified from Lee et al. (2006) is in the left panel. The dashed lines link bases that can form H-bonds in a tertiary folding. The structure of wheat 5SRNA indicates the C loop in domain β is important in binding to the RPL5 as discussed in the text. The right panel (dotted line) indicates the wheat RPL5 protein gene models (reference sequence from Kang et al., 2011) and illustrates the gene-level haplotypes discussed in the text for TraesCS2A02G296000, TraesCS2B02G312400, TraesCS2D02G293900, TraesCS5B02G374800.2, and TraesCS5D1G474800LC (see main text for details of the identification process). The TraesCS2D02G293900 gene model has the C-terminal 84 amino acids deleted and is most likely a pseudogene. The significance of the inserted three amino acids at positions 25–27 is unclear. The alignment of the five wheat RPL5 gene models indicates that they are largely separated into either chromosome 2A/2B or chromosome 5B/5D protein haplotypes with the chromosome 2D entry showing a large deletion. At the genome level, the single nucleotide polymorphism (SNP)-based haplotypes were limited to introns and thus useful for tracking the gene region but none of the within-genome variation between homologous genes has been captured in the wheat varieties accessible in the genome viewer DAWN. The red line highlights the region predicted to bind the section of 5SRNA highlighted in a red dashed line in the left panel. The breaks in the rows of *s indicate amino acid changes in the alignments, and these generally define the chromosome 2A/2B or chromosome 5B/5D protein haplotypes.

The 5S rRNA component of the rRNA is synthesized independent from the 18S, 5.8S, and 26S rRNA (see Figure 1C and Supplementary Figure 2), and in model systems, the ribosomal L5 protein (RPL5) has been shown to be an important factor for the correct assembly of 5S rRNA into the 60S subunit (Figure 4) together with RPL11, into a feature of the 60S subunits called the central protuberance (CP). The CP feature is close to the peptidyl-transferase center (PTC) and GTPase-associating center sites (Armache et al., 2010). Although the exact function of 5S rRNA is not well defined, Kang et al. (2011) have shown that in wheat the effects of salt, drought, and/or freezing stress caused a rapid accumulation of the RPL5 (TaL5) transcript in seedling leaves. It is thus possible that the variation in the formation of the 5S rRNA–RPL5 complex as a result of quantitative changes and qualitative variation in the RPL5 amino acid sequence (gene-level haplotypes, Figure 4) could modify the translation properties of the ribosome to be more suited to the stress conditions.

## The Ribosomal Proteins Assembled Into Ribosomes

The structure of the wheat ribosome has been determined at the 5.5-Å angstrom level of resolution (Armache et al., 2010), and we have used the respective accession numbers of the 40S and 60S RPs in this structure to identify the gene models in the wheat genome. The cryo-EM technology combined with modeling utilizing yeast and other microbe ribosome structures allowed Armache et al. (2010) to compile a consensus structure of a translating wheat ribosome in which RP α-helices were observed as rod-like densities and β-sheets were assigned by smooth surfaces. The authors noted that α-helix pitch and β-sheet strand separation could not be determined. The wheat RPs identified as gene models using the information from Armache et al. (2010) mostly identified homologous gene models at loci on each of the A, B, and D genomes (IWGSCrefSeqver1, IWGSC, 2018). A key criterion for gene models was that they were all highly transcribed in the standard tissues, grain, leaf, roots, stem, and spike (Supplementary Table 1). The translated gene models were run in Phyre2 (Kelley et al., 2015) to identify RP domains and were then cross-referenced to InterPro (Blum et al., 2020) for annotating the RP-encoding genes in the wheat genome followed by matching them to Traes ID codes for gene models in the reference wheat genome. Intron– exon structures were checked for consistency with the aligned RNA-seq available in the Apollo genome viewer. The annotations in Supplementary Table 1 identified 25 groups of Traes IDs for the 40S subunit RPs and 37 groups of Traes IDs for 60S subunit RPs with the respective reference gene (plus UniProt ID), usually from rice, also indicated for each group. The Traes gene model alignments from a given group or subgroup showed high levels of conservation as a foundation for assessing the low levels of variation that defined gene haplotypes at the amino acid level (concept developed further below). Proteome level confirmation that the gene models coded for wheat proteins was obtained for eight of the 25 groups assigned to 40S subunits and for 13 of the 37 groups assigned to 60S subunits. In the case of the RPL6 group, these entries were checked in detail because of an interest in the change in quantity of the protein designated as RPL6, in response to water stress (Islam et al., 2020), and it was found that the two amino acid positions that differentiated TraesCS6B02G225600 and TraesCS6D02G190100 could be identified in the respective peptide maps. The grouping and naming of RPs in Supplementary Table 1 was ambiguous in some cases due to the presence of shared RNA-binding domains and the historical aspects surrounding RP nomenclature (Ban et al., 2014); the assembly of RPs in Supplementary Table 1 is intended to provide a sequence-based point of reference for the wheat RPs. Only 10 of the wheat gene models in Supplementary Table 1 have “LC” added in the IWGSC reference, indicating “low confidence”; for these gene models, the intron– exon structures and reading frames were curated manually to ensure that the models included in Supplementary Table 1 were in fact HC. For the 40S subunit RPs, Armache et al. (2010) included the RP, RACK1 protein C kinase as one of the proteins in their 3D wheat ribosome assembly, and the respective wheat genome Traes IDs are provided. It is possible that in light of the wheat germination study by Smailov et al. (2020) of the phosphorylation of RPS6 protein by RPS6 kinase, TaS6K1 (AK451448), that the RPS6 kinase may be a more appropriate model than RACK1 protein C kinase with respect to identifying a relevant 40S subunit RP-Traes IDs in the wheat genome. For completeness, both sets of Traes IDs are indicated in Supplementary Table 1, in addition to the broader regulator of translation, TOR, which is responsible for activating the RPS6 kinase (Smailov et al., 2020).

**FIGURE 5:**
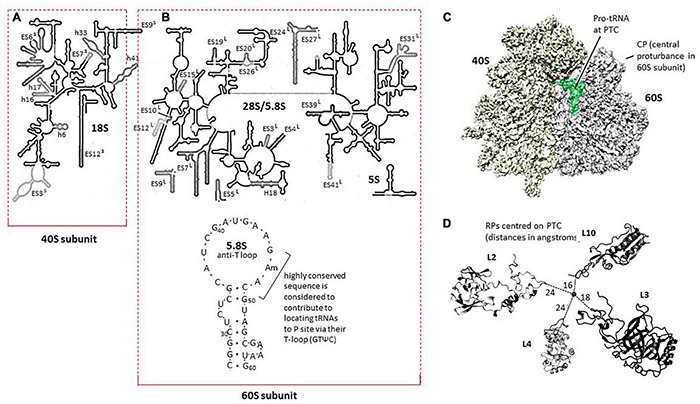
**(A)** Map of the 18S rRNA in the 40S ribosome subunit modified from Armache et al. (2010; doi.org/10.1073/pnas.1009999107). The h and ES loops in the rRNA molecule are discussed in the text. **(B)** Map of the 26S rRNA in the 60S ribosome subunit modified from Armache et al. (2010, doi.org/10.1073/pnas.1009999107). The wheat 5.8S rRNA molecule shown was based on Mackay et al. (1980). **(C)**. The 3D representation of the translating wheat ribosome with the Proline-tRNA at the peptidyl-transferase center (PTC) in place Armache et al. (2010, doi.org/10.1073/pnas.1009999107). The central protuberance in the 60S subunit is a standard landmark for the 60S subunit. **(D)** Relationship between RPL2, RPL3, RPL4, and RPL10 modeled in the wheat ribosome PTC from Armache et al. (2010)Supplementary Data in doi.org/10.1073/pnas.100999910.

The framework for defining the wheat RPs in the context of the detailed 3D compilation of the translating ribosome is provided in Figure 5. In Figures 5A,B, the maps of the wheat rRNA molecules are shown with the ES annotations, indicating the extension segments to RNA molecules relative to bacterial rRNA reference sequences (Armache et al., 2010). Figures 5A,B also indicates the codes for some of the helical structures of the rRNA molecules since these are sites for binding RPs.

The wheat 5.8S rRNA (Figure 5B) is assembled into the 60S subunit near the PTC, whereas in model systems, it has been suggested that the highly conserved GAACG in the anti-T loop (see Figure 5B) contributes to engaging incoming tRNA-amino acid entities at the PTC through the equally highly conserved (complementary) sequence GTΨC in tRNAs (Nishikawa and Takemura, 1974; Mackay et al., 1980). The 5.8S rRNA has also been argued, in model systems, to contribute to the inclusion of the translation elongation factors involved in the peptide translocation process for peptide synthesis (Elela et al., 1994).

Although model systems have shown that rRNA segments configured at the PTC provide ribozyme activity for catalyzing peptide bond formation, and that RPs are not strictly required for this chemical reaction (reviewed in De la Cruz et al., 2015), it is evident that, within the context of the biology of the cell, RPs are critical (reviewed in De la Cruz et al., 2015). The RPs are required for the many steps in forming and stabilizing the ribosome complex in order to ensure the efficient translation of mRNA. Utilizing the gene models documented in Supplementary Table 1, variation in wheat RPs can now be compiled to provide gene haplotypes (as more broadly considered by Wilhelm et al., 2013) that document variation between proteins from homologous loci on the A, B, and D genomes and SNP variation at the DNA sequence level, to indicate the potential functional markers for associating particular RP variants with phenotypic attributes. The alignments of the RPs within the groups and subgroups in Supplementary Table 1 indicate six groups of RPs in the 40S subunit and 11 in the 60S subunit and show no variation in their amino acid sequence within the IWGSCrefseqver1 genome sequence, whereas the remainder provides the amino acid variation that can be considered as gene haplotypes. The variation complements the variation presented earlier for nucleolin (Supplementary Figure 1) and fibrillarin (Figure 3), two abundant proteins important for the infrastructure of the nucleolus and rRNA production and the TBP, Rrn3, UAF30, and Rrp5 gene models for establishing the initiation complex to start rRNA synthesis.

Specific examples of the variation between proteins from homologous loci on the A, B, and D genomes are provided below for RPS6 because it is historically significant, RPL6 as an example of an RP that was responsive to water stress in wheat (Islam et al., 2020), and RPL2, RPL3, RPL4, and RPL10 because of their particular importance in forming the PTC (Figure 5D).

### RPS6

One of the earliest examples of phosphorylation of an RP was for RPS6 (reviewed in Biever et al., 2015) and, based on studies in model systems, it is one of the RPs interacting with rRNA transcripts during their processing in the nucleolus (Bernstein et al., 2004) to form mature rRNAs. Although the functions of RPS6 have not been clearly defined, its phosphorylation has been used as a marker for the coordinated phosphorylation and activation of RPS6 kinase, S6K1, and activation of the translation initiation factor eIF4B (Holz et al., 2005). Importantly, the activation of RPS6 kinase also reflects the activity of a central regulator of cell proliferation and growth in eukaryotic cells, target of rapamycin (TOR). In wheat, the TOR–S6K1 signaling pathway has been shown to be a key step in GA-induced digestion of starch in the germinating wheat grain for seedling growth (Smailov et al., 2020).

**FIGURE 6:**
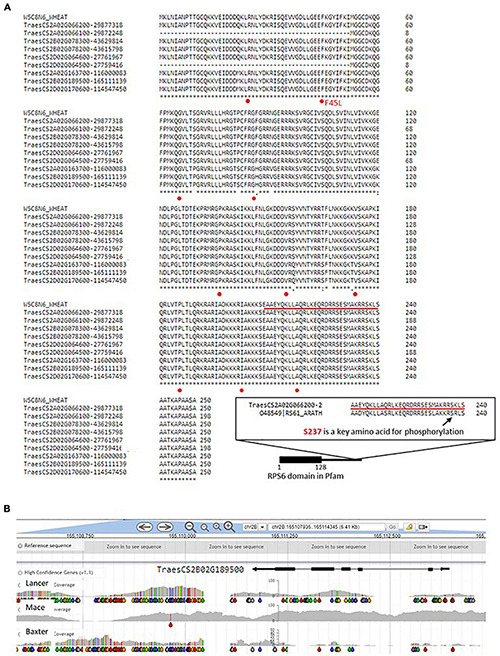
Alignments for wheat RPS6 gene models at the amino acid level. **(A)** The first six entries in the alignments are on the short arms of chromosomes 2A, 2B, and 2D, and the lower three entries are on the respective long arms. CLUSTAL omega (Sievers et al., 2011; https://www.ebi.ac.uk/Tools/services/web/) was used to carry out the alignments using standard parameters. The Traes IDs are for IWGSCrefseqver1 and a hyphen separates the coordinate position of the gene model; the gaps in the entries TraesCS2A02G066100 and TraesCS2DG0264500 are the result of gaps in the IWGSCrefseq-ver1 assembly. The insert is the alignment of a small section of amino acid sequence from TraesCS2A02G066200 and an Arabidopsis RPS6 (O48549) referred to in the text in relation to the Serine237. This section is also underlined in the main sequence and is shown in location relative to the highly conserved RPS6 domain (black box). The single nucleotide polymorphisms (SNPs) indicated in panel **(B)** (below) at the genome level are indicated with red dots where they occur in the CDS and in only one case the SNP changed the code for the amino acid (F45L, indicated in red). **(B)** Comparison of the SNP profiles from two representative wheat varieties showing strikingly different haplotypes in the region of the wheat chromosome 2B locus for RPS6 as defined in Pfam (Orengo et al., 2020) at positions 1–128. The gray areas indicate the variable genome coverage of the available sequence data and the colored “drops” identify positions in the sequence that are uniformly changed from that of the reference Chinese Spring genome sequence (orange = change to G; red = change to T; green = change to A; blue = change to C). The SNP analysis was possible using DAWN (Watson-Haigh et al., 2018; http://crobiad.agwine.adelaide.edu.au/dawn/jbrowse/). * means identical amino acid in that position across the genes.

The alignments shown in Figure 6 indicate that only eight amino acid differences are found between the predicted proteins. However, at the genome level, wheat varieties analyzed in DAWN show a clear haplotype difference at the genome level that is evident in the example shown for TraesCS2B02G189500 at location 165111139 on chromosome 2B. Among the nine SNPs at the genome level in the CDS, only one caused an amino acid change, F45L, which was not represented in the differences found between the A, B, and D homologs within the reference wheat genome *per se*. None of the other Traes-RPS6 models in Figure 6 showed SNPs in the CDS and in light of the polyploid nature of wheat, indicates that a major change, with an unknown phenotype, such as F45L in one RPS6 gene is extensively buffered by no changes in the other gene models. A similar situation is indicated for all the amino acid variations identified in Figure 6A where the amino acid changes for homologous genes on the long arm of 2A, 2B, and 2D (lower three entries in the alignment) for example are not found in the short arm loci. The missing sequences from TraesCS2A02G066100 and TraesCS2D02G064500 are due to gaps in the respective genome assemblies of chromosomes 2A and 2D, based on the inspection of the published reference genome of Chinese Spring. It is of interest that the phosphorylation of the serine at position 237 (S237) in Arabidopsis is closely linked to the light–dark cycle in the environment and the internal circadian rhythm of the plant (Enganti et al., 2018), and since the respective amino acid sequence section of RPS6 can be clearly identified in the wheat gene model (see insert Figure 6A), it adds interest to the phosphorylation of RPS6 as a marker in wheat as discussed above. The increase in S237 phosphorylation in the dark to light transition correlates with the increased loading of ribosomes onto mRNA to form polysomes in Arabidopsis (Enganti et al., 2018).

Considering the well-established nature of RPS6 as a marker for the status of cell proliferation and growth of the plant, in many model systems, the clear haplotype at the genome level differentiating two successful varieties, Mace and Lancer (Figure 6B) suggest that the genome level variation provides a useful fingerprint for associating genome changes with phenotypic variation. The details of the SNP scoring in Figure 6B using DAWN (Watson-Haigh et al., 2018) relative to the reference wheat genome assembly are provided in the legend of the figure.

### RPL6

**FIGURE 7:**
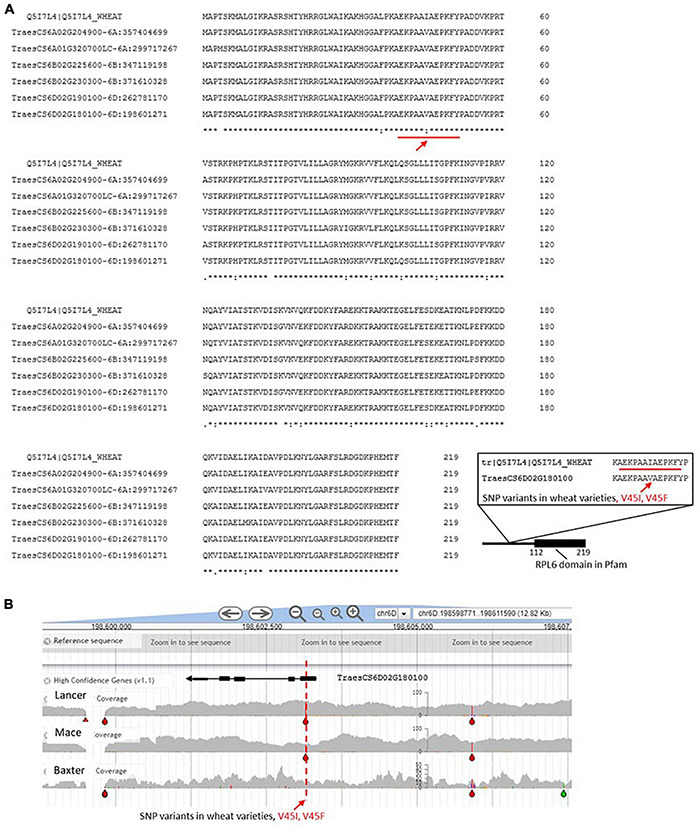
**(A)** RPL6 wheat gene models. See legend Figure 3 for details of the identification process. The red line refers to a section of the amino acid sequence that is also represented in the inset to emphasize that the V45L and V45F changes have been captured in the wheat varieties examined in DAWN [see panel **(B)**]. **(B)** Screen for single nucleotide polymorphism (SNPs) in the RPL6 region of chromosome 6D. The gray areas indicate the variable genome coverage of the available sequence data and the colored “drops” identify positions in the sequence that are uniformly changed from that of the reference Chinese Spring genome sequence (orange = change to G; red = change to T; green = change to A; blue = change to C). The SNP analysis was possible using DAWN (Watson-Haigh et al., 2018; http://crobiad.agwine.adelaide.edu.au/dawn/jbrowse/). The red dashed lines emphasizes the V45L and V45F changes in the amino acid sequence shown in Figure 6A within an otherwise conserved CDS.

In an extensive iTRAQ proteomic analysis of water stress administered at the reproductive stage in wheat, Islam et al. (2020) found that ribosomal protein L6 (RPL6) was one of the few RPs that responded to the stress and showed a 4.2-fold increase (*p*-value of 0.008). The RPL6 identified in the proteome study was TraesCS6D02G190100, which is one of a pair of RPL6 proteins on chromosome 6D (Figure 7); pairs of RPL6 proteins also exist at homologous loci on chromosomes 6A and 6B (Figure 7). The response to water stress was considered rapid because the expressional change was detected before head emergence, immediately after the drought stress was imposed. RPL6s are among key proteins for controlling and enhancing protein synthesis and have been studied in several plant species in response to different environmental stresses including high and low temperatures, salinity, and water deficit. In rice, the overexpression of RPL6 resulted in tolerance to moderate (150 mM) to high (200 mM) levels of NaCl (Sahi et al., 2006; Moin et al., 2020). In addition, 50S RPL6 was upregulated after 48 h of drought stress in maize (Pei et al., 2019). Salt stress can result in modification of protein synthesis, and it has been observed that in RPL6 transgenic rice plants the upregulation of genes encoding RPs in plants under stressed conditions can lead to efficient reconstruction of protein-synthesizing machinery in cells under stress without compromising the growth and development. The RPL6 protein family members are also highly upregulated in heat-primed wheat plants compared with the non-heat-primed plants (Wang et al., 2016).

Although RPL6 is highly conserved and identification through homology to model systems was unambiguous, we note that 24 variable positions in the amino acid sequence are evident within the hexaploid wheat entries (Figure 7). These variable positions provided sufficient resolution between the wheat gene models to allow the pairs of genes on chromosome 6D to be distinguished (different haplotypes at the amino acid level) and specifically assign TraesCS6D02G190100 to be the gene that was upregulated at the protein level as a result of water stress early in the head development (Islam et al., 2020). The example shows that one mechanism for selecting RP sequence variants to make up the pool of ribosomes in wheat includes the possibility of quantitative changes in the level of expression.

Inspection of wheat varieties available in the DAWN genome viewer for SNPs in the RPL6 CDS detected a G to T change leading to an altered amino acid (V45K) relative to the reference sequence. The arrow in Figure 7 indicates that at this position a V45I variation is detected from including a non-reference wheat sequence (from the UniProt database) in the alignment. The biological consequence of this polymorphism remains to be determined.

### RPL2

Relatively few studies are available for the biological effects of modifying RPL2 levels of expression or mutational changes since most studies focus on the plastid RPL2 protein. For the 60S subunit RPL2, Ludwig and Tenhaken (2001) found that the transient loss of RPL2 expression in soybean challenged with a fungal infection correlated with a loss of protein synthesis activity but improved tolerance to the infection. The authors argued that the reduced protein synthesis activity in infected cells provided a short-term response to reduce the capacity of the infecting fungus to utilize host cell resources and allowed the host cell to produce its own defense molecules. Interestingly, the RPL2 gene models in wheat are one of the few examples of absolute conservation of amino acid sequence (Supplementary Figure 5); hence, there exists no option to recruit suitable, preexisting sequence variants of RPL2 from the genes in wheat. This leaves only a quantitative change in the expression as a mechanism for a change in ribosome translation attributes driven by RPL2. At the genomic level, no SNPs are found within the CDS when wheat varieties were inspected.

### RPL3

**FIGURE 8:**
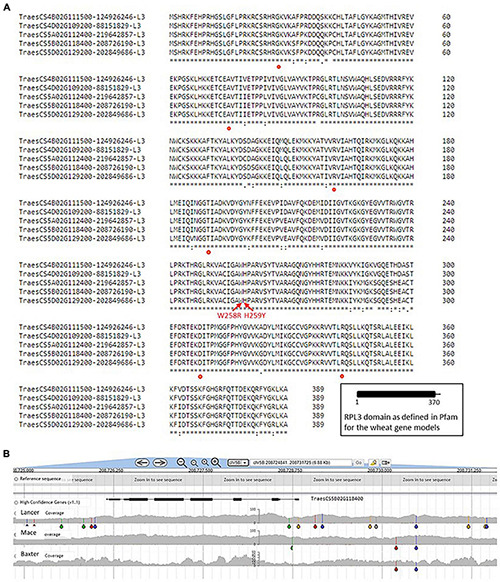
RPL3 gene models in wheat. **(A)** See legend Figure 3 for details of the identification process. Locations of differences in amino acid sequence within a single genome are indicated by the absence of a *. The red dots indicate the locations of mutations associated with medical conditions in humans and are thus predicted to have a phenotype in wheat if they were found to occur. The two red arrows highlight mutations at positions W258R and H259Y in the tomato RPL3 that provided an improved tolerance to *F. graminearum* in transgenic tobacco. **(B)** Single nucleotide polymorphism (SNP) variation in the genome region around the RPL3 gene on chromosome 5B, even though variation in the CDS has not been captured in the set of wheat varieties available in the DAWN viewer used to generate the image. The gray areas indicate the variable genome coverage of the available sequence data and the colored “drops” identify positions in the sequence that are uniformly changed from that of the reference Chinese Spring genome sequence (orange = change to G; red = change to T; green = change to A; blue = change to C). The SNP analysis was possible using DAWN (Watson-Haigh et al., 2018; http://crobiad.agwine.adelaide.edu.au/dawn/jbrowse/).

In contrast to RPL2, the RPL3 gene models do show some sequence variation (Figure 8) although a screen for SNP variation in the DAWN genome viewer for wheat varieties indicated that no variation in the CDS has been captured in the set of varieties examined (Supplementary Figure 6). The RPL3 is located in the PTC of wheat ribosomes (see Figure 5D) and reduced levels of RPL3 in transgenic *Nicotiana* (Popescu and Tumer, 2004) correlated with reductions in cell number, stunting, and inhibition of lateral root growth. Precursor rRNA levels (32S, see Figure 1C) were elevated in the transgenic plants consistent with an early engagement of RPL3 as part of the generation of rRNA. Mutations identified in human RPL3 (G27N, A75V, R161W, T189M, D308N(V), and R343W) are associated with medical conditions (Thorolfsdottir et al., 2017; Ganapathi et al., 2020) but have not been identified as variable positions in wheat RPL3 to date. The findings in human RPL3 do serve to indicate that variation in this gene can be explored in terms of fine-tuning the translation process to particular phenotypic requirements such as tolerance to stress conditions. The mutations at positions W258R and H259Y in the tomato RPL3 provided an improved tolerance to *Fusarium graminearum* in transgenic tobacco (Safipoor-Afshar et al., 2007) due to a reduced sensitivity to the trichothecen mycotoxin DON in the host plants, and even though these are not variable positions in the survey of wheat genome SNPs, they provide interesting targets. Di and Tumer (2005) reported that a transgenic construct carrying a truncated RPL3 missing the C-terminal region also conferred increased tolerance to DON in transgenic tobacco plants.

### RPL4

The wheat RPL4 gene models show a low level of sequence divergence, which is spread across the proteins domains that define the gene model (Supplementary Figure 7). In Arabidopsis, the mutation G73R (numbering follows Supplementary Figure 7) modifies the phenotype extensively to generate a plant with narrow leaves, abnormal numbers of cotyledons, short roots, and short hypocotyls. Although the G73R mutation has undesirable phenotypic consequences, it is able to suppress deleterious mutations (Horiguchi et al., 2011; Kakehi et al., 2015), which may be an advantage in certain situations of a genetic modification pipeline to suppress deleterious mutations transfers to wheat.

### RPL10

**FIGURE 9:**
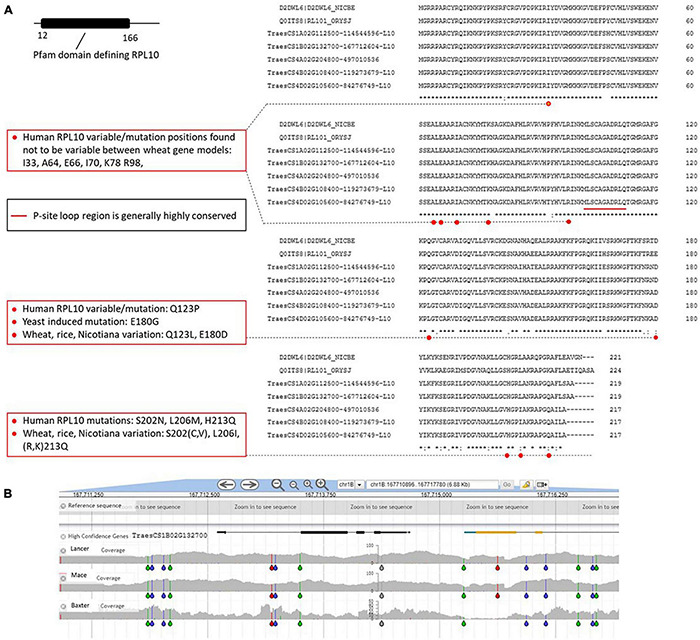
**(A)** RPL10 wheat gene models. See legend Figure 3 for details of the identification process. The red dots indicate the locations of the amino acid changes found in human, wheat, rice tobacco, and yeast shown in the respective boxes to the left of the alignment, discussed in the text. In some positions, breaks in the *s indicating differences within the homologous wheat genes align correspond to red dots (for example Q123L) indicating the change is also found in humans and other eukaryotes as defined in the boxes to the left of the alignments. The red line indicates the P-site loop region that is important in the catalytic site (PTC, see Figure 5). **(B)** Single nucleotide polymorphism (SNP) variation in the genome region around the RPL10 gene on chromosome 1B, even though variation in the CDS has not been captured in the wheat varieties examined (exemplar shown). The gray areas indicate the variable genome coverage of the available sequence data and the colored “drops” identify positions in the sequence that are uniformly changed from that of the reference Chinese Spring genome sequence (orange = change to G; red = change to T; green = change to A; blue = change to C). The SNP analysis was possible using DAWN (Watson-Haigh et al., 2018; http://crobiad.agwine.adelaide.edu.au/dawn/jbrowse/).

The location of RPL10 is also near the PTC (Figure 5D) in microorganism, plants, and animals and extends from the CP (Figure 5C) to the PTC/GTP-ase center *via* the P-site loop region of RPL10. The P-site loop is in the middle of RPL10 at positions 102–112 and is a conserved amino acid sequence. The P-site is argued to be required for the conformational changes within the ribosome that are associated with the elongation cycle of the translation process for synthesizing peptide chains (reviewed in Pollutri and Penzo, 2020) in model systems. In the P-site loop region of the wheat RPL10, no differences exist among the gene models (Figure 9). Consistent with its fundamental importance in biology, variation is limited to 41 positions (including the very C-terminal end) across humans to plants in the 224 amino acid sequence; among 33 induced mutations in yeast that could be located in the wheat sequence, 17 were lethal, whereas others such as the change in E (glutamic acid) at position 180 significantly reduced yeast growth. In a mutation study in Arabidopsis (Falcone-Ferreyra et al., 2013), the altering of three copies of the RPL10 gene resident in the genome indicated that compensation between copies occurred and expression differed between tissues. These Arabidopsis studies and studies in human (Klauck et al., 2006; reviewed in Pollutri and Penzo, 2020) and *Nicotiana benthamiana* (Ramu et al., 2020) are consistent with the more general model that variation in essential RPs, such as RPL10, can moderate the translation activity of ribosomes to preferentially accept certain mRNAs and/or undertake non-ribosomal level regulation of transcription and signal transduction.

## Discussion

The cytoplasmic ribosomes constitute the central RNA–protein complex responsible for synthesizing new proteins in wheat cells, and studies in model organisms have developed the concept that diversity in the composition of ribosomes can be linked to phenotypic diversity (reviewed in Pollutri and Penzo, 2020). In plants, the observations relating to clusters of rRNA unit types that can be co-regulated in a tissue-specific manner support the concept of tissue-specific ribosome subpopulations differing in their functional attributes and contributing to responses to environmental challenges (Tulpová et al., 2020; Sims et al., 2021). The ribosomes from wheat-germ extracts were among the early eukaryotic cell-free systems for translating mRNA, and the analyses summarized in the present study provide a foundation for characterizing populations of ribosomes with a range of functional attributes. Specifically, the model argues that certain ribosomes can become predominant in situations of stress for preferentially translating mRNAs to generate proteins better suited to contributing to the survival of the cell in situations of, for example, water stress (Martinez-Seidel et al., 2020). This level of structural variation in the population of ribosomes would interact with the well-established variation in the translation machinery of the cell in response to stresses with respect to the engagement of initiation factors and elongation factors involving a translational regulator TOR in wheat (Smailov et al., 2020), and more broadly across plants and animals. The outputs from integrating the networks that determine the translation of populations of mRNA in a cell can be visualized experimentally using ribosome profiling which is based on sequencing of ribosome protected mRNA fragments, combined with total RNA-seq data, to provide an estimate of the efficiency of the utilization of particular mRNAs (Lei et al., 2015). Maize seedlings under water stress (Lei et al., 2015) provided evidence for changes in the sequence profile of translated mRNA and changes in transcription *per se* that correlated with a water stress response.

In wheat, an additional variable relates to the discovery of ribosome-inactivating proteins (tritins) that have different specificities and cofactor requirements depending on whether they are from seed or other tissues (Massiah and Hartley, 1995). The tritins in wheat comprise a family of 17 genes and inspection of IWGSC ver2.1 (Zhu et al., 2021), using the UniProtKB—Q07810 sequence, indicated six gene models expressed predominantly in grain tissue and at a lower level in root tissue in a cluster on chromosome 5B. An additional eight gene models on 5B expressed only in root tissue but at a lower level. Three tritin gene models were identified on chromosome 5A expressed at a relatively level in grain and root tissue, and no significant hits could be identified on chromosome 5D. Two gene models were closely linked and moderately expressed in grain and root and on a genome segment not assigned to a chromosome. The rRNA N-glycosidase activity associated with tritins can preferentially depurinate highly conserved 26S rRNA SRL sequences (sarcin–ricin loop, AGUACGAGAGGA) required for elongation factor engagement at the PTC, in the ribosomes from invading pathogens (Fernandez-Puentes and Vazquez, 1977). This activity can therefore provide a novel approach for developing disease resistance in wheat. Similarly, the RPL3 protein is also located at the PTC and mutations at positions W258R and H259Y, or deletions of the C-terminal region, in the RPL3 gene provided an improved tolerance to *F. graminearum* in transgenic tobacco (Di and Tumer, 2005; Safipoor-Afshar et al., 2007). The improved resistance was due to a reduced sensitivity to DON produced by *F. graminearum* and thus provides a target for breeding tolerance to this challenging pathogen in wheat. This approach could complement the success in detoxifying trichothecene mycotoxins such as DON using the glutathione-*S*-transferase gene encoded by the Fhb7 locus (Wang et al., 2020).

Mutations and variation, in model systems, in RPL2, RPL4, and RPL10 proteins that are also located at the PTC support the concept of the rRNA–protein complex as possible targets for modifying the phenotype of the wheat plant. In the case of RPL10, some of the variable positions between the wheat homologous loci corresponded to the position of mutations in the human RPL10 that associated with disease phenotypes (ribosomopathies; Pollutri and Penzo, 2020). The example of RPL6 discussed in this manuscript also brings into play the ribosome independent functions of many RPs where it has been argued in model systems that stress can initiate the translocation of RPL6 from the nucleolus to the nucleoplasm where it can interact with chromatin histone H2A and alter cell biology (Yang et al., 2019).

Inspection of variation among the wheat RP gene models in Supplementary Table 1 generates a picture of single amino acid changes (gene haplotypes) that provide wheat with a flexible pool of RPs and hence pools of ribosomes with unique functional attributes for changing the balance of mRNAs translated in particular environmental conditions. The variation in RPs interface with the variation documented in the rRNA that is exemplified by nucleolar dominance, to generate populations of ribosomes with unique compositions suitable in certain physiological conditions of the cell. An extensive literature exists to describe the effects of ABA on ribosome attributes (Grill and Himmelbach, 1998; Guo et al., 2011; Zhang et al., 2012; Li et al., 2021) and would be expected to reflect the engagement of the protein synthesis machinery in the higher level control networks within the cell.

## Author Contributions

RA conceived, analyzed/interpreted the data, and drafted the manuscript. SI and PW carried out the proteome studies and contributed to writing of the manuscript. All authors contributed to the article and approved the submitted version.

## Conflict of Interest

The authors declare that the research was conducted in the absence of any commercial or financial relationships that could be construed as a potential conflict of interest.

## Publisher’s Note

All claims expressed in this article are solely those of the authors and do not necessarily represent those of their affiliated organizations, or those of the publisher, the editors and the reviewers. Any product that may be evaluated in this article, or claim that may be made by its manufacturer, is not guaranteed or endorsed by the publisher.

## Footnotes

1
http://plants.ensembl.org/index.html

2
http://apollo.tgac.ac.uk/Wheat_IWGSC_WGA_v1_0_browser

3
http://www.sbg.bio.ic.ac.uk/∼phyre2/html/page.cgi?id=index

4
http://pfam.xfam.org/search

5
http://www.ebi.ac.uk/interpro/search/sequence-search
