# Supplementary material for: Integrating Wheat Nucleolus Structure and Function: Variation in the Wheat Ribosomal RNA and Protein Genes
Source: Front Plant Sci. 2021 Dec 24;12:686586. doi: 10.3389/fpls.2021.686586 (PMC8739226; doi:10.3389/fpls.2021.686586)
Supplement: Supplementary file 3 [file Presentation_1.pdf]

**Supplementary S1- variation in the amino acid sequence for the wheat nucleolin gene family**

Variation in the amino acid sequence for the wheat nucleolin gene family. In the Traes ID's the 2A, 2D, 7A, 7B, 7D designate the chromosomal location of the gene model

Assignment of the helicase activity to the glycine-rich region is based on Tong et al., (1997). The wheat homologs on chromosome 2A, 2D has four more exons than the 7A, 7B and 7D homologs and were consistent with the definitions of nucleolins in Arabidopdsis (Pontvianne et al.,., 2007) and Oryza sativa. The wheat 2A, 2D, 7A, 7B and 7D all had the same 3-D fold signature (RNA binding, c6r5kH in Phyre2) as the rice and Arabidopsis nucleolins.

A

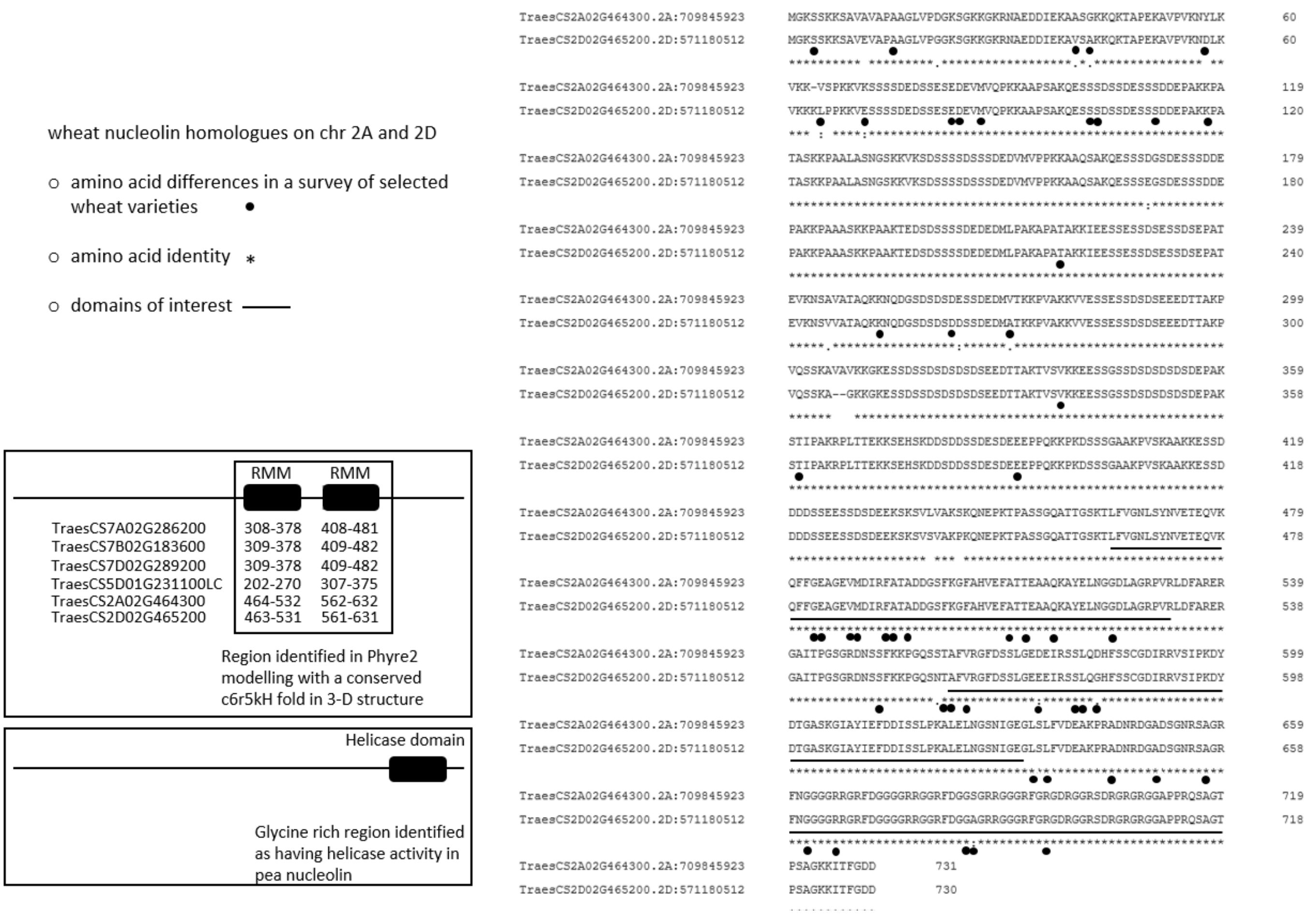


B

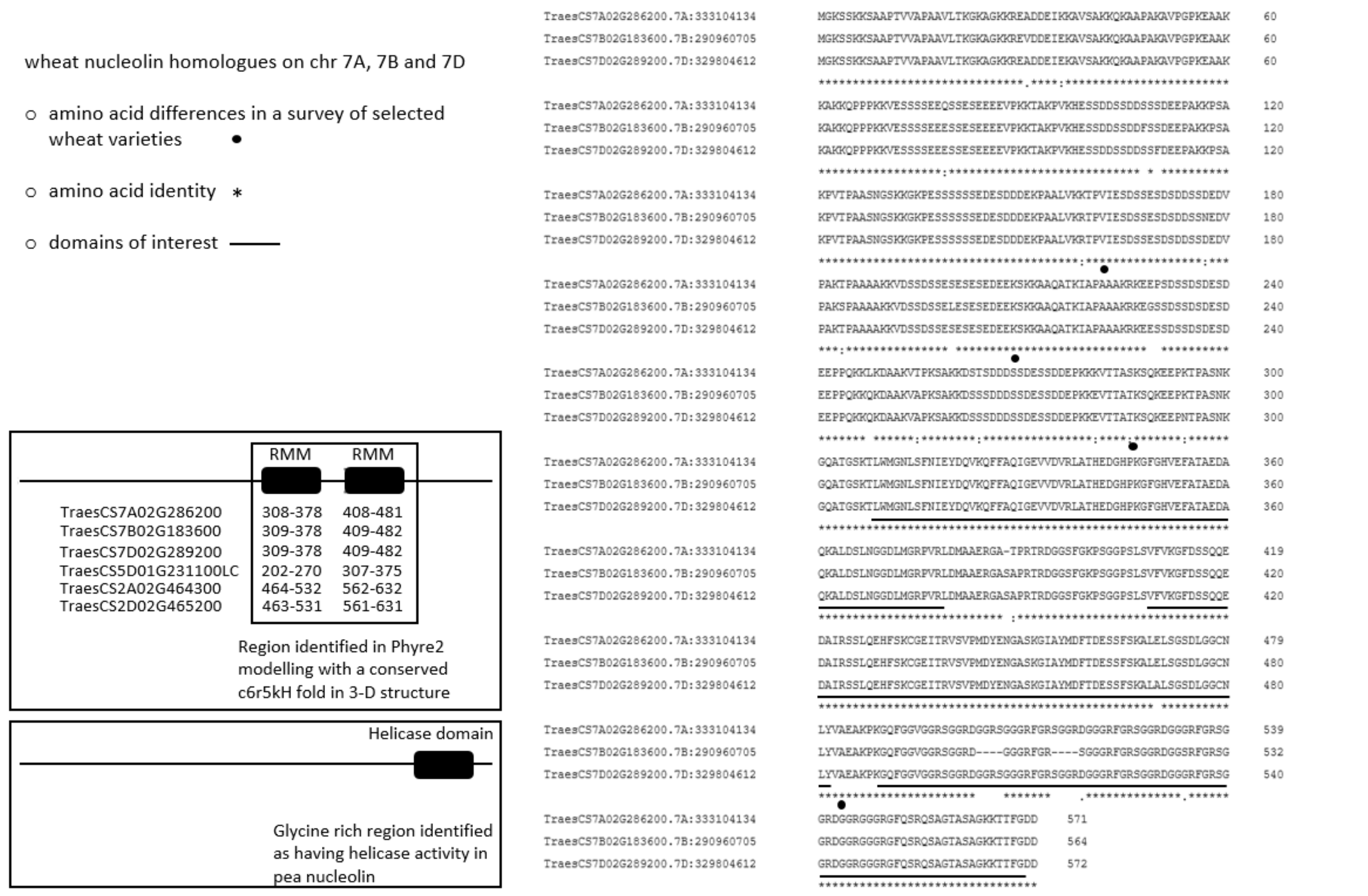


Figure S1. The 2A and 2D nucleolin genes are aligned in part A, and the 7A, 7B and 7D genes in part B

## Supplementary S2- wheat 5S gene repeating units on 1AS, 1BS and 1DS

Sequence of the short wheat 5S gene repeating units on 1AS, 1BS and 1DS, with the gene shown in black and the TATA box is indicated (part A in Figure below). The long 5S units are represented in tandem arrays on chromosomes 5AS, 5BS and 5DS are 485 – 504 bp (representative sequence AF150605) and have a TTGGGCGAGAGTAGT tandem duplication of positions 60 to 75 of the gene; these genes are not represented in the 5S transcripts

The predicted components of the transcription complex for generating 5S rRNA in wheat are shown in part B of the Figure below)

• PLATZ (reference maize, GRMZM2G006585; Wang et al., 2018) wheat family of homologs (low confidence), TraesCS2A02G448300, TraesCS2B02G469000, TraesCS6A02G264400, TraesCS6D02G250500

• TATA TBP wheat homologs, TraesCS1B02G151700, TraesCS5A02G022000, TraesCS5B02G018500, TraesCS5D02G027800

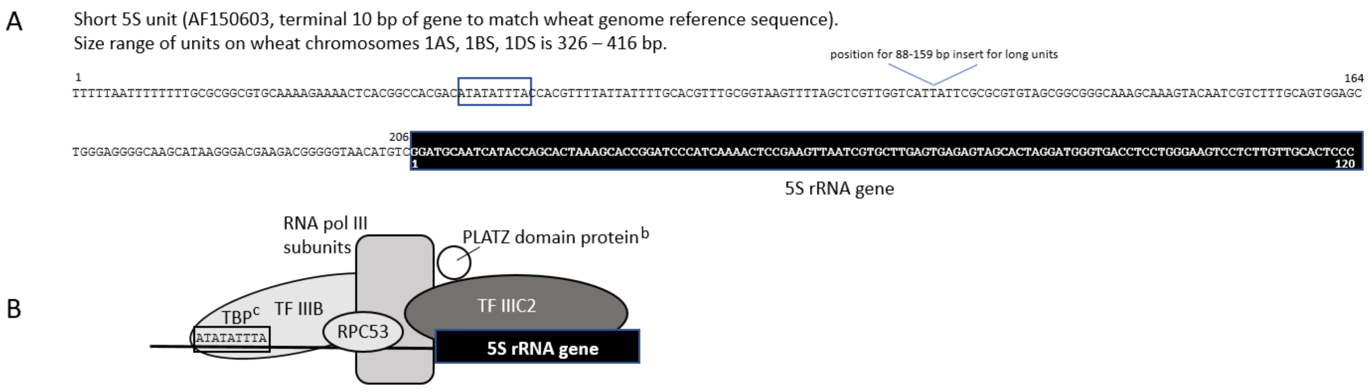


Figure S2. The transcription of 5S rRNA in wheat.

## Supplementary S3. The initiation complex for RNA polymerase I

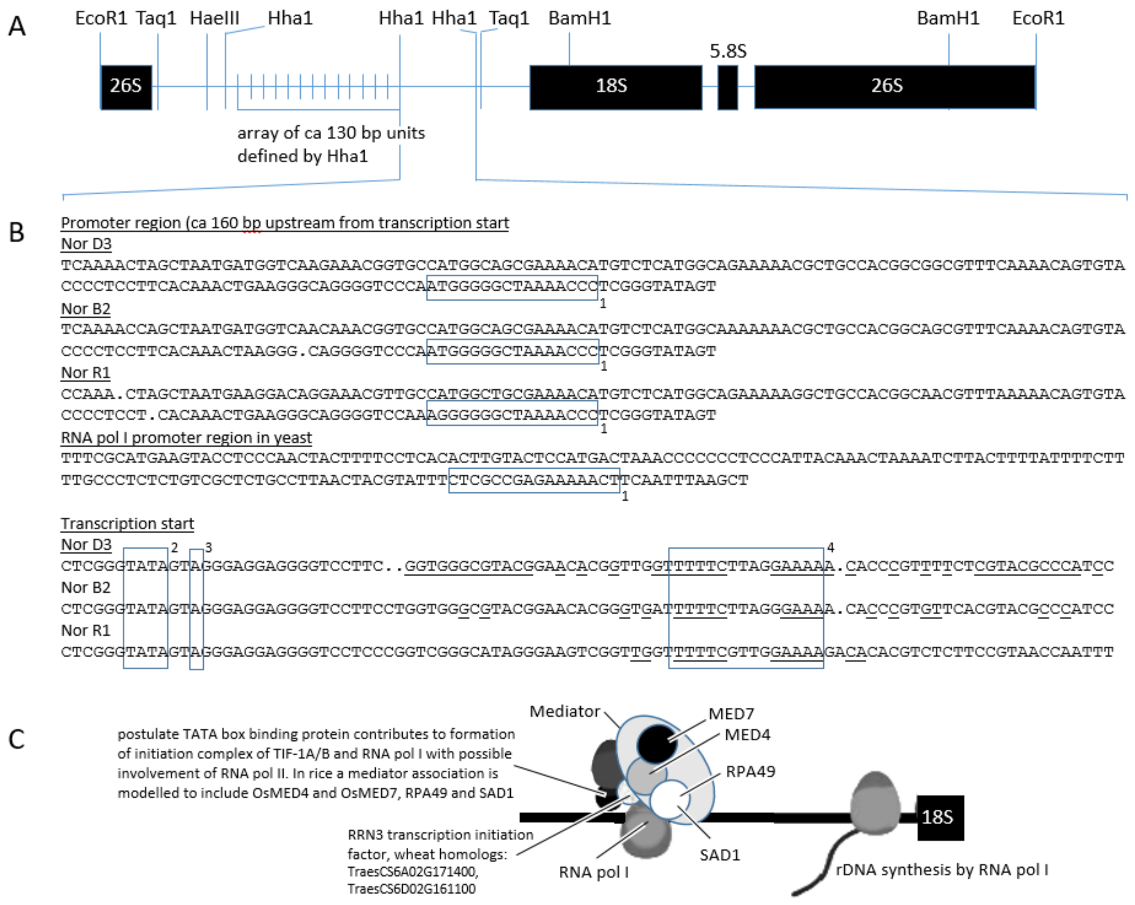


Figure S3. The initiation complex for RNA polymerase I

A. Overview of the wheat rDNA rpeat unit as reported in Appels and Dvorak (1982)

B. Promoter region ([1]) with signature sequences such as TATA shown in boxes ([2] and [3]) as well as the transcription start ([4])

C. Postulated initiation complex for wheat RNA polymerase 1

## Supplementary S4: Rrp5 genome region polymorphisms:

The DAWN viewer (Watson-Haigh et al., 2018; http://crobiad.agwine.adelaide.edu.au/dawn/jbrowse/) for SNP variation relative to the reference genome of Chinese Spring and a selected set of wheat varieties including two of the 10 genome sequences, Mace and Lancer

(Walkowiak et al., 2020) to indicate the variation found. The grey areas indicate the variable genome coverage of the available sequence data and the colored “drop” identify positions in the sequence that are uniformly changed from that of the reference Chinese Spring genome sequence (orange=change to G; red=change to T; green=change to A; blue=change to C). The SNP analysis was possible using DAWN

Major SNP haplotypes for can be readily identified

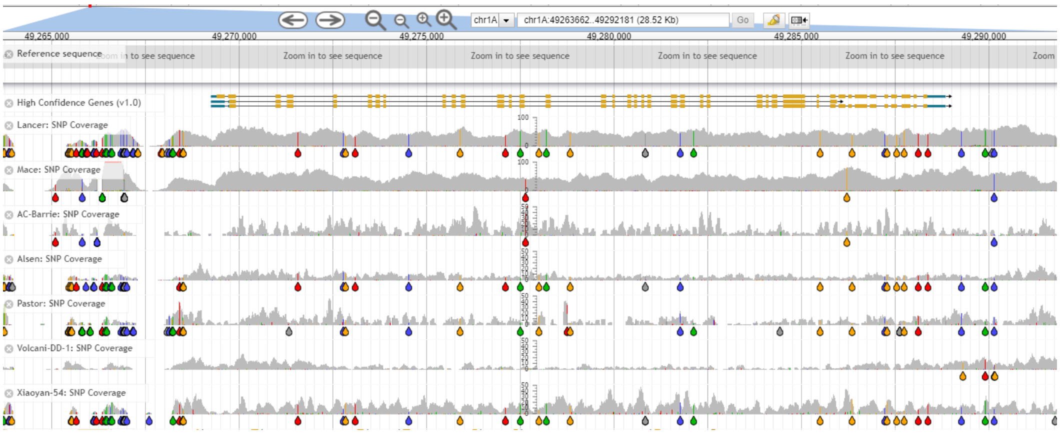


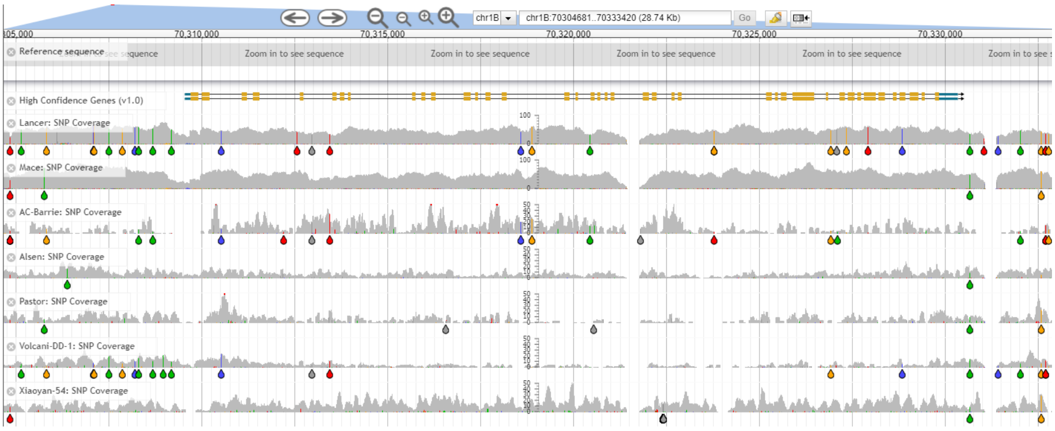


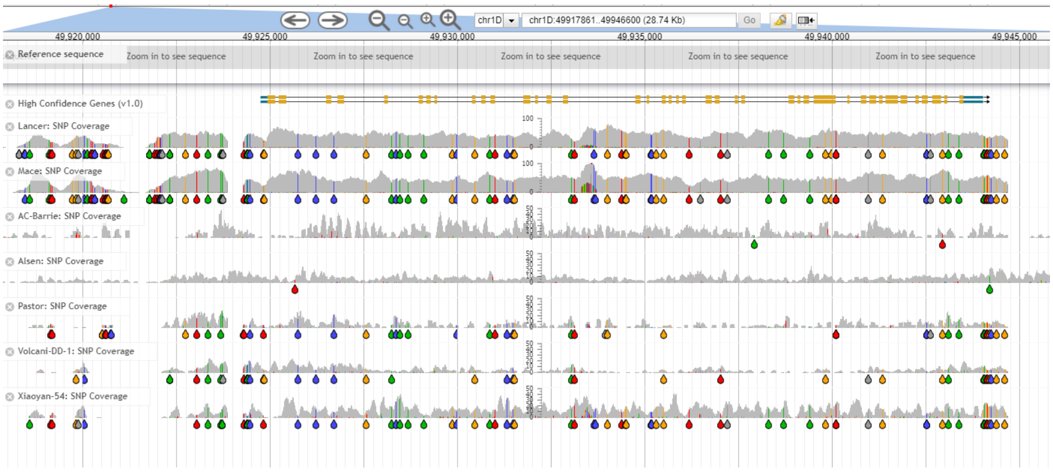


## Supplementary S5: RPL2 CDS alignments and genome region polymorphisms

```
TraesCS4B02G128700-168565690-L2      MGRVIRAQRKGAGSVFKSHTHHRKGPARFRSLDFGERNGYLKGVVTDVIHDPGRGAPLAK      60
TraesCS4D02G123700-108051182-L2      MGRVIRAQRKGAGSVFKSHTHHRKGPARFRSLDFGERNGYLKGVVTDVIHDPGRGAPLAK      60
TraesCS5A02G082800-108279551-L2      MGRVIRAQRKGAGSVFKSHTHHRKGPARFRSLDFGERNGYLKGVVTDVIHDPGRGAPLAK      60
TraesCS5B02G088900-114425373-L2      MGRVIRAQRKGAGSVFKSHTHHRKGPARFRSLDFGERNGYLKGVVTDVIHDPGRGAPLAK      60
TraesCS5D02G095400-104625542-L2      MGRVIRAQRKGAGSVFKSHTHHRKGPARFRSLDFGERNGYLKGVVTDVIHDPGRGAPLAK      60
                                     ************************************************************

TraesCS4B02G128700-168565690-L2      VTFRHPFRYKHQKELFVAAEGMYTGQFVYCGRRATLSVGNVLPLRSVPEGGVICNVEHHV      120
TraesCS4D02G123700-108051182-L2      VTFRHPFRYKHQKELFVAAEGMYTGQFVYCGRRATLSVGNVLPLRSVPEGGVICNVEHHV      120
TraesCS5A02G082800-108279551-L2      VTFRHPFRYKHQKELFVAAEGMYTGQFVYCGRRATLSVGNVLPLRSVPEGGVICNVEHHV      120
TraesCS5B02G088900-114425373-L2      VTFRHPFRYKHQKELFVAAEGMYTGQFVYCGRRATLSVGNVLPLRSVPEGGVICNVEHHV      120
TraesCS5D02G095400-104625542-L2      VTFRHPFRYKHQKELFVAAEGMYTGQFVYCGRRATLSVGNVLPLRSVPEGGVICNVEHHV      120
                                     ************************************************************

TraesCS4B02G128700-168565690-L2      GDRGVFARASGDYAIVISHNPDNGTSRIKLPSGAKKIVPSSCRAMIGQVAGGGRTEKPML      180
TraesCS4D02G123700-108051182-L2      GDRGVFARASGDYAIVISHNPDNGTSRIKLPSGAKKIVPSSCRAMIGQVAGGGRTEKPML      180
TraesCS5A02G082800-108279551-L2      GDRGVFARASGDYAIVISHNPDNGTSRIKLPSGAKKIVPSSCRAMIGQVAGGGRTEKPML      180
TraesCS5B02G088900-114425373-L2      GDRGVFARASGDYAIVISHNPDNGTSRIKLPSGAKKIVPSSCRAMIGQVAGGGRTEKPML      180
TraesCS5D02G095400-104625542-L2      GDRGVFARASGDYAIVISHNPDNGTSRIKLPSGAKKIVPSSCRAMIGQVAGGGRTEKPML      180
                                     ************************************************************

TraesCS4B02G128700-168565690-L2      KAGNAYHKYRVKRNSWPKVRGVAMNPVEHPHGGGNHQHIGHASTVRRDAPPGQKVGLIAA      240
TraesCS4D02G123700-108051182-L2      KAGNAYHKYRVKRNSWPKVRGVAMNPVEHPHGGGNHQHIGHASTVRRDAPPGQKVGLIAA      240
TraesCS5A02G082800-108279551-L2      KAGNAYHKYRVKRNSWPKVRGVAMNPVEHPHGGGNHQHIGHASTVRRDAPPGQKVGLIAA      240
TraesCS5B02G088900-114425373-L2      KAGNAYHKYRVKRNSWPKVRGVAMNPVEHPHGGGNHQHIGHASTVRRDAPPGQKVGLIAA      240
TraesCS5D02G095400-104625542-L2      KAGNAYHKYRVKRNSWPKVRGVAMNPVEHPHGGGNHQHIGHASTVRRDAPPGQKVGLIAA      240
                                     ************************************************************

TraesCS4B02G128700-168565690-L2      RRTGRLRGQAAASAAKADKAT      261
TraesCS4D02G123700-108051182-L2      RRTGRLRGQAAASAAKADKAT      261
TraesCS5A02G082800-108279551-L2      RRTGRLRGQAAASAAKADKAT      261
TraesCS5B02G088900-114425373-L2      RRTGRLRGQAAASAAKADKAT      261
TraesCS5D02G095400-104625542-L2      RRTGRLRGQAAASAAKADKAT      261
                                     *********************
```

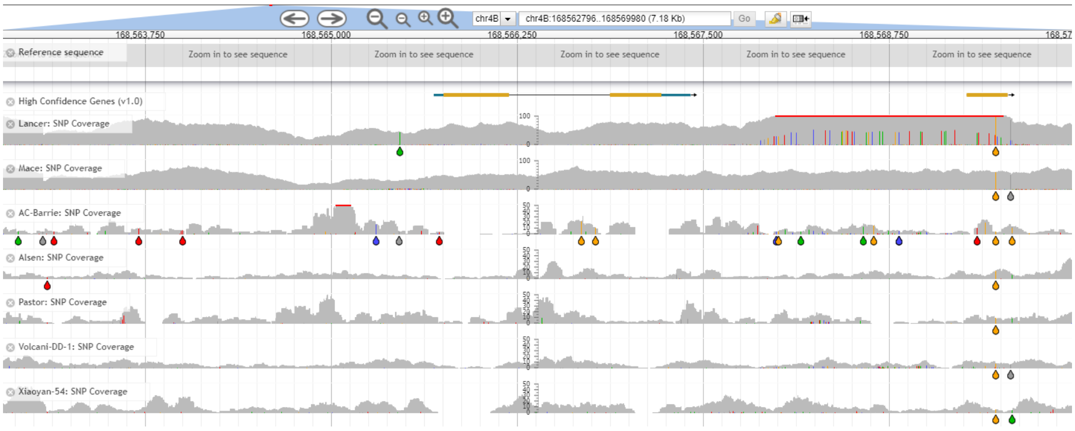


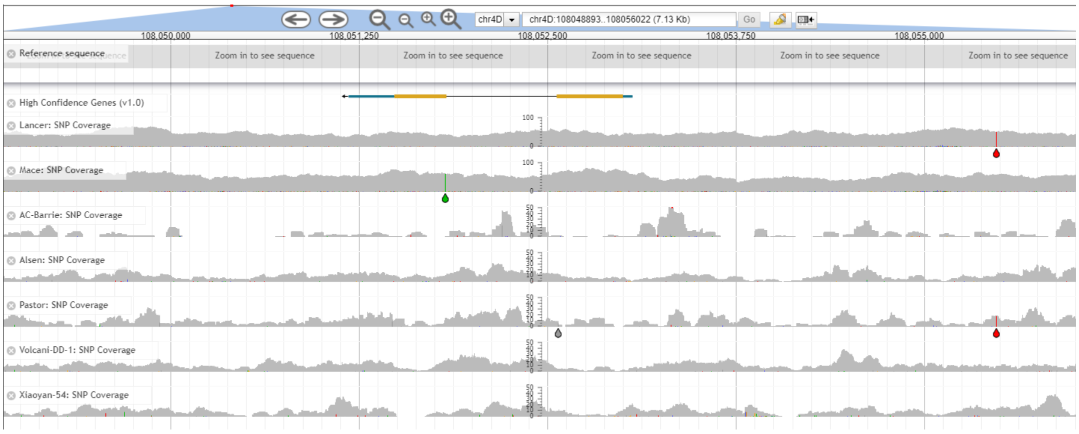

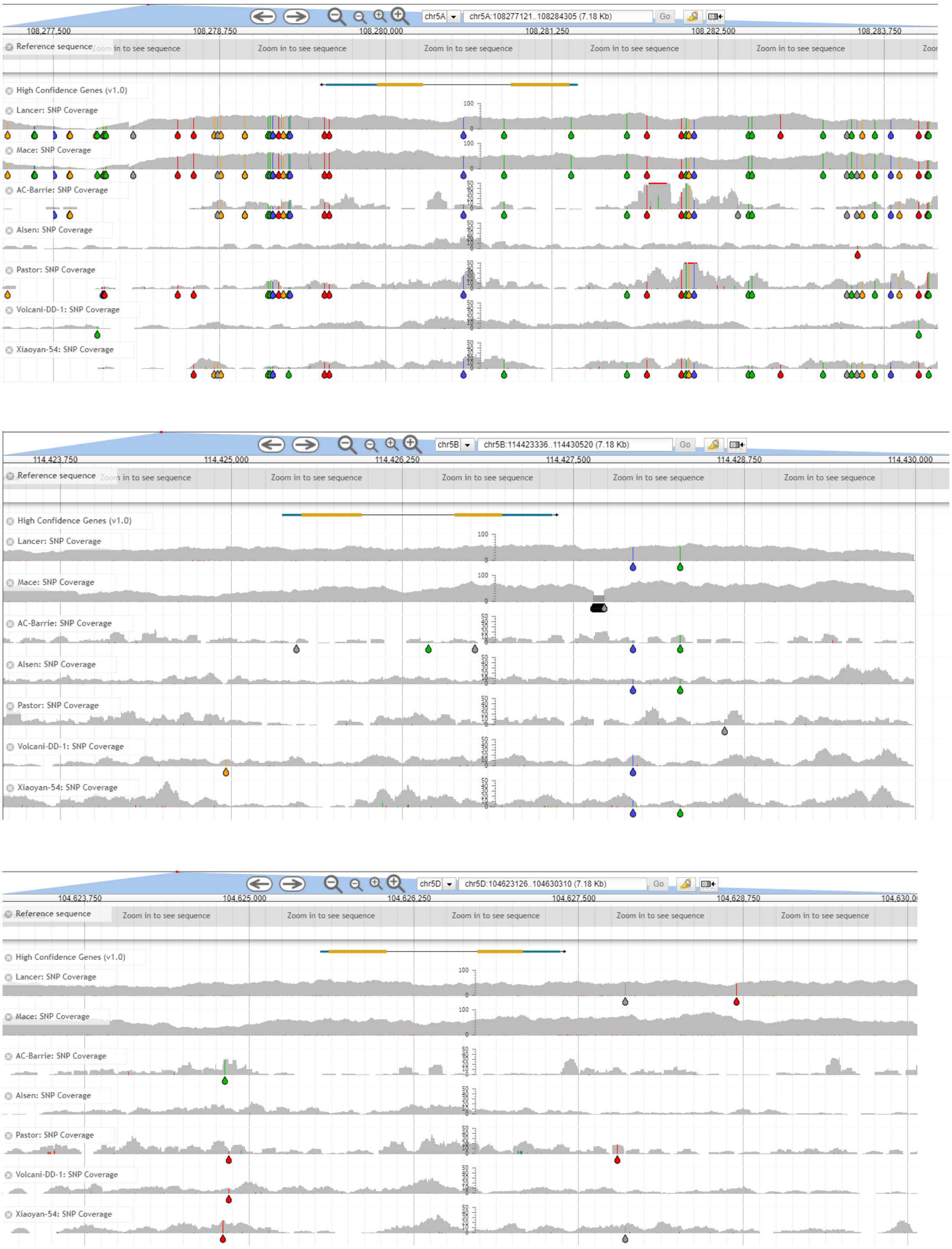


**Supplementary S6: RPL3 genome region polymorphisms**

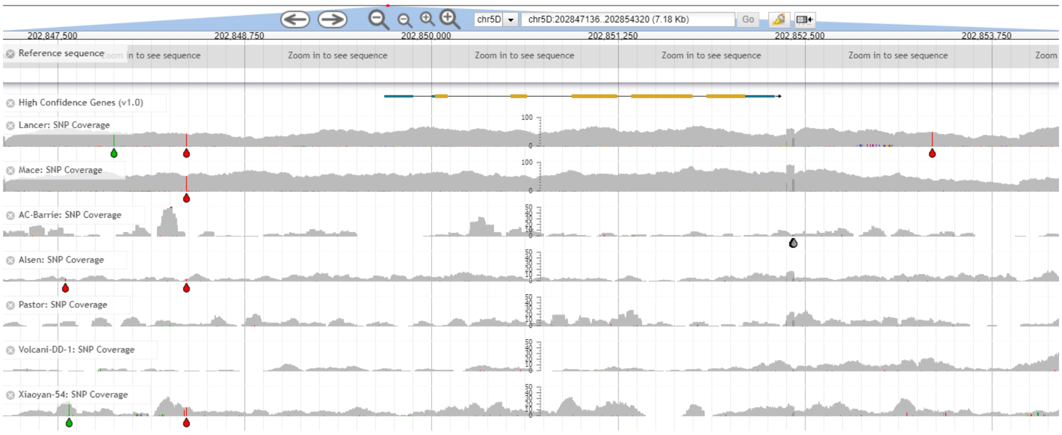
chr5D
chr5D:202847136..202854320 (7.18 Kb)
Go
202,847,500
202,848,750
202,850,000
202,851,250
202,852,500
202,853,750
Reference sequence
Zoom in to see sequence
High Confidence Genes (v1.0)
Lancer: SNP Coverage
Mace: SNP Coverage
AC-Barrie: SNP Coverage
Alsen: SNP Coverage
Pastor: SNP Coverage
Volcani-DD-1: SNP Coverage
Xiaoyan-54: SNP Coverage

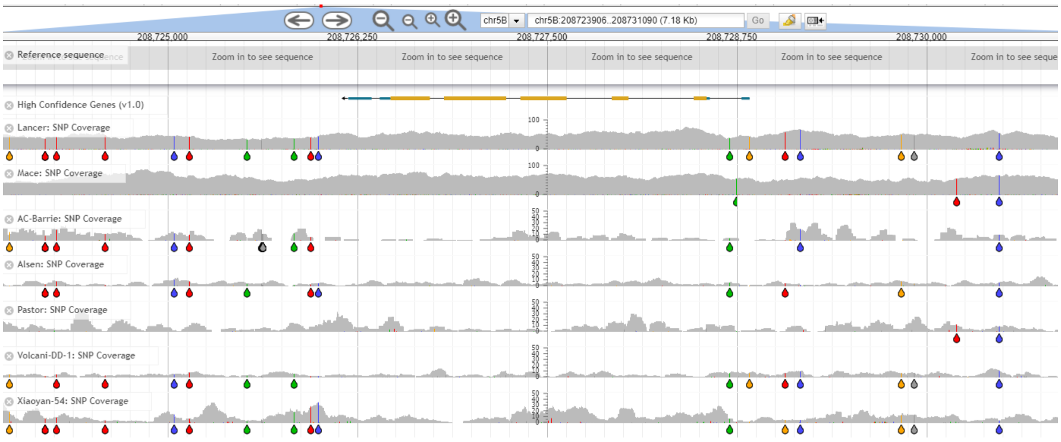
chr5B
chr5B:208723906..208731090 (7.18 Kb)
Go
208,725,000
208,726,250
208,727,500
208,728,750
208,730,000
Reference sequence
Zoom in to see sequence
High Confidence Genes (v1.0)
Lancer: SNP Coverage
Mace: SNP Coverage
AC-Barrie: SNP Coverage
Alsen: SNP Coverage
Pastor: SNP Coverage
Volcani-DD-1: SNP Coverage
Xiaoyan-54: SNP Coverage

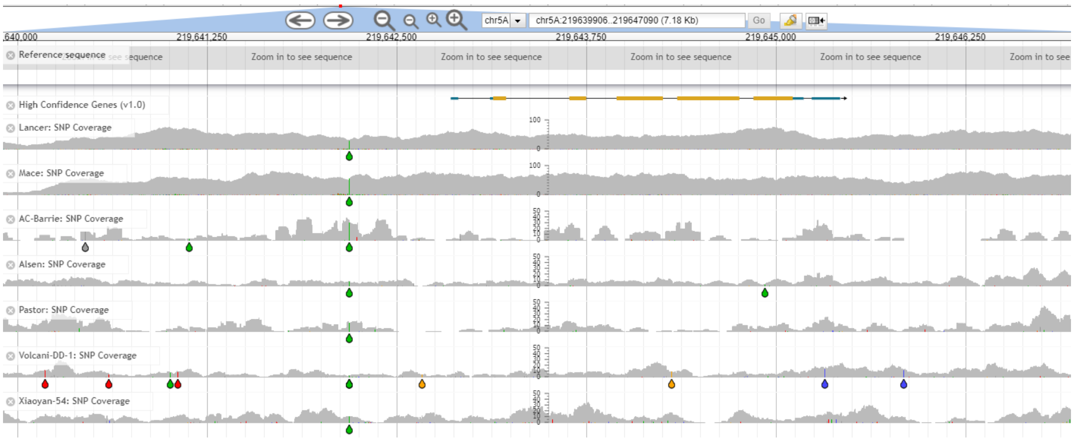
chr5A
chr5A:219639906..219647090 (7.18 Kb)
Go
219,641,250
219,642,500
219,643,750
219,645,000
219,646,250
Reference sequence
Zoom in to see sequence
High Confidence Genes (v1.0)
Lancer: SNP Coverage
Mace: SNP Coverage
AC-Barrie: SNP Coverage
Alsen: SNP Coverage
Pastor: SNP Coverage
Volcani-DD-1: SNP Coverage
Xiaoyan-54: SNP Coverage

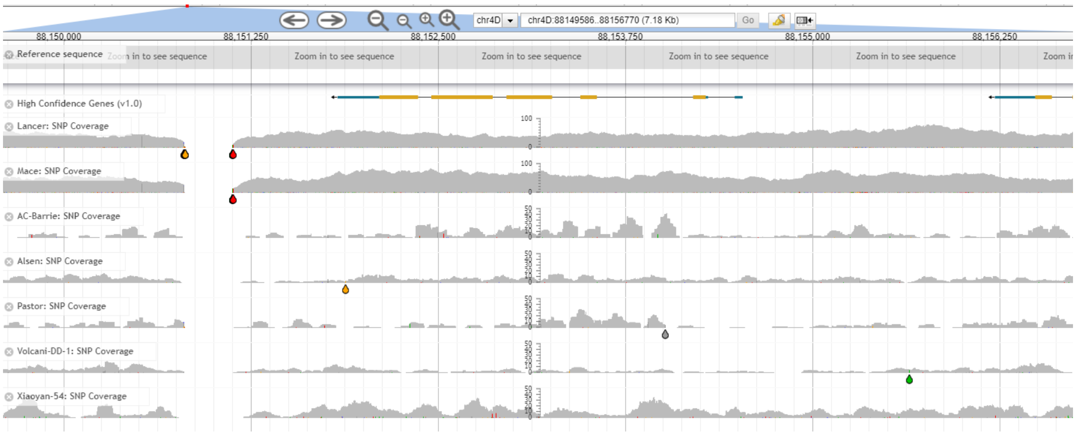


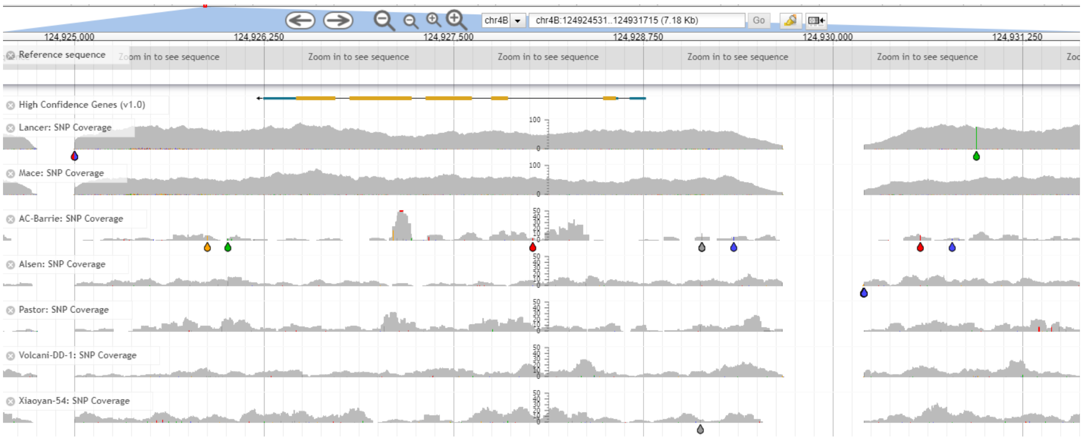


## Supplementary S7: RPL4 CDS and genome region alignments

```
TraesCS1A02G354400-537352530-L4-moderate    MATTARPLVSVKALDGDMPTDAAGVPMPHVMKAPIRPDVITFVHRLVSCNSRQPYAVSRK    60
TraesCS1D02G264200-360093847-L4-high        MATTARPLVSVKALDGDMATDAAGVPMPHVMKAPIRPDVITFVHRLVSCNSRQPYAVSRK    60
TraesCS5D02G446300-496588497-L4             MATTARPLVSVKALDGDMATDAAGVPMPHVMKAPIRPDVITFVHRLVSCNSRQPYAVSRK    60
TraesCS1B02G274700-482164469-L4-high        MATTARPLVSVKALDGDMATDAAGVPMPHVMKAPIRPDVITFVHRLVSCNSRQPYAVSRK    60
TraesCS5B02G442100-615200228-L4             MATTARPLVSVKALDGDMATDAAGVPMPHVMKAPIRPDVITFVHRLLSCNSRQPYAVSRK    60
                                            ****************** ***************************:*************

TraesCS1A02G354400-537352530-L4-moderate    AGHQTSAESWGTGRAVSRIPRVGGGGTHRSGQGAFGNMCRGGRMFAPTRIWRKWHRRVNI    120
TraesCS1D02G264200-360093847-L4-high        AGHQTSAESWGTGRAVSRIPRVGGGGTHRAGQGAFGNMCRGGRMFAPTRIWRKWHRRVNV    120
TraesCS5D02G446300-496588497-L4             AGHQTSAESWGTGRAVSRIPRVGGGGTHRAGQGAFGNMCRGGRMFAPTRIWRKWHRRVNV    120
TraesCS1B02G274700-482164469-L4-high        AGHQTSAESWGTGRAVSRIPRVGGGGTHRAGQGAFGNMCRGGRMFAPTRIWRKWHRRVNV    120
TraesCS5B02G442100-615200228-L4             AGHQTSAESWGTGRAVSRIPRVGGGGTHRAGQGAFGNMCRGGRMFAPTRIWRKWHRRVNV    120
                                            *****************************:*****************************:

TraesCS1A02G354400-537352530-L4-moderate    RLRRVAVASALAATAVPAIVTARGHRIESVPEFPLVVSDSAEGIEKTSQAVKVLKQLGAY    180
TraesCS1D02G264200-360093847-L4-high        RLRRVAVASALAATAVPAIVTARGHRIESVPEFPLVVSDSAEGIEKTAQAIKVLKQLGAY    180
TraesCS5D02G446300-496588497-L4             RLRRVAVASALAATAVPAIVTARGHRIESVPEFPLVVSDSAEGIEKTAQAIKVLKQLGAY    180
TraesCS1B02G274700-482164469-L4-high        RLRRVAVASALAATAVPAIVTARGHRIESVPEFPLVVSDSAEGIEKTSQAIKVLKQLGAY    180
TraesCS5B02G442100-615200228-L4             RLRRVAVASALAATAVPAIVTARGHRIESVPEFPLVVSDSAEGIEKTAQAIKVLKQLGAY    180
                                            ***********************************************:**:*********

TraesCS1A02G354400-537352530-L4-moderate    ADADKAKDSVGIRPGKGKMRNRRYINRKGPLIVYATEGSKIVKAFRNLPGVDVANVERLN    240
TraesCS1D02G264200-360093847-L4-high        ADAEKAKDSVGIRPGKGKMRNRRYINRKGPLIVYGTEGSKIVKAFRNLPGVDVANVERLN    240
TraesCS5D02G446300-496588497-L4             ADAEKAKQSVGIRPGKGKMRNRRYINRKGPLIVYGTEGSKIVKAFRNLPGVDVANVERLN    240
TraesCS1B02G274700-482164469-L4-high        ADAEKAKDSVGIRPGKGKMRNRRYINRKGPLIVYGTEGSKIVKAFRNLPGVDVANVERLN    240
TraesCS5B02G442100-615200228-L4             ADAEKAKESVGIRPGKGKMRNRRYINRKGPLIVYGTEGSKIVKAFRNLPGVDVANVERLN    240
                                            ***:***:**************************.*************************

TraesCS1A02G354400-537352530-L4-moderate    LLDLAPGGHLGRFVIWTESAFKKLDEVYGSFEASSSKKKGFVLPRPKMTNADLGRLINSD    300
TraesCS1D02G264200-360093847-L4-high        LLDLAPGGHLGRFVIWTESAFKKLDEVYGSFEASSSKKKGFVLPRPKMTNADLGRLINSD    300
TraesCS5D02G446300-496588497-L4             LLDLAPGGHLGRFVIWTESAFKKLDEVYGSFEASSSKKKGFVLPRPKMTNADLGRLINSD    300
TraesCS1B02G274700-482164469-L4-high        LLDLAPGGHLGRFVIWTESAFKKLDEVYGSFEASSSKKKGFVLPRPKMTNADLGRLINSD    300
TraesCS5B02G442100-615200228-L4             LLDLAPGGHLGRFVIWTESAFKKLDEVYGSFEASSSKKKGFVLPRPKMTNADLGRLINSD    300
                                            ************************************************************

TraesCS1A02G354400-537352530-L4-moderate    EVQSVVKPINKEVKRREARKNPLKNAAAVLKLNPYFGTARRMAVLAEAARVKARKDKINS    360
TraesCS1D02G264200-360093847-L4-high        EVQSVVKPINKEVKRREARKNPLKNAAAVLKLNPYFGTARRMAVLAEAARVKARKEKINS    360
TraesCS5D02G446300-496588497-L4             EVQSVVKPINKEVKRREARKNPLKNAAAVLKLNPYFGTARMMAVLAEAARVKARKEKINS    360
TraesCS1B02G274700-482164469-L4-high        EVQSVVKPINKEVKRREARKNPLKNAAAVLKLNPYFGTARRMAVLAEAARVKARKEKINS    360
TraesCS5B02G442100-615200228-L4             EVQSVVKPINKEVKRREARKNPLKNAAAVLKLNPYFGTARRMAVLAEAARVKARKEKINS    360
                                            **************************************** **************:****

TraesCS1A02G354400-537352530-L4-moderate    KRTKLSVEEASKIKAAGKAWYQTMISDSDYMEFDVFSKWLGVSQ    404
TraesCS1D02G264200-360093847-L4-high        KRTKLSAEEASKIKAAGKAWYQTMISDSDYTEFDVFSKWLGVSQ    404
TraesCS5D02G446300-496588497-L4             KRTKLSAEEASKIKAAGKAWYQTMISDSDYTEFDVFSKWLGVSQ    404
TraesCS1B02G274700-482164469-L4-high        KRTKLSAE-ASKIKAAGKAWYQTMISDSDYTEFDVFSKWLGVSQ    403
TraesCS5B02G442100-615200228-L4             KRTKLSAE-ASKIKAAGKAWYQTMISDSDYTEFDVFSKWLGVSQ    403
                                            ******.* ********************* *************
```
